# Graphene Synthesis Techniques and Environmental Applications

**DOI:** 10.3390/ma15217804

**Published:** 2022-11-04

**Authors:** Qaisar Abbas, Pragati A. Shinde, Mohammad Ali Abdelkareem, Abdul Hai Alami, Mojtaba Mirzaeian, Arti Yadav, Abdul Ghani Olabi

**Affiliations:** 1 Sustainable Energy & Power Systems Research Centre, RISE, University of Sharjah, Sharjah 27272, United Arab Emirates; 2 School of Engineering, Computing & Physical Sciences, University of the West of Scotland, Paisley PA1 2BE, UK; 3 Chemical Engineering Department, Minia University, Minya 61519, Egypt; 4 Faculty of Chemistry and Chemical Technology, Al-Farabi Kazakh National University, Al-Farabi Avenue, 71, Almaty 050012, Kazakhstan; 5 School of Engineering, Newcastle University, Newcastle upon Tyne NE1 7RU, UK; 6 Mechanical Engineering and Design, School of Engineering and Applied Science, Aston University Aston Triangle, Birmingham B4 7ET, UK

**Keywords:** graphene synthesis, two-dimensional material, chemical vapor deposition, exfoliation, environmental applications

## Abstract

Graphene is fundamentally a two-dimensional material with extraordinary optical, thermal, mechanical, and electrical characteristics. It has a versatile surface chemistry and large surface area. It is a carbon nanomaterial, which comprises sp^2^ hybridized carbon atoms placed in a hexagonal lattice with one-atom thickness, giving it a two-dimensional structure. A large number of synthesis techniques including epitaxial growth, liquid phase exfoliation, electrochemical exfoliation, mechanical exfoliation, and chemical vapor deposition are used for the synthesis of graphene. Graphene prepared using different techniques can have a number of benefits and deficiencies depending on its application. This study provides a summary of graphene preparation techniques and critically assesses the use of graphene, its derivates, and composites in environmental applications. These applications include the use of graphene as membrane material for the detoxication and purification of water, active material for gas sensing, heavy metal ions detection, and CO_2_ conversion. Furthermore, a trend analysis of both synthesis techniques and environmental applications of graphene has been performed by extracting and analyzing Scopus data from the past ten years. Finally, conclusions and outlook are provided to address the residual challenges related to the synthesis of the material and its use for environmental applications.

## 1. Introduction

**Figure 1:**
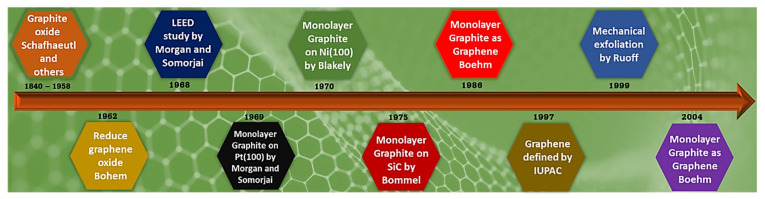
Timeline of historical development of graphene.

The scientific community around the globe working on material research is overwhelmed by the research focused on carbon-based nanomaterials, and the emphasis is particularly on the fabrication, characterization, and real-word applications of extremely thin carbon films, where graphene tends be the thinnest and therefore most explored carbon-based nanomaterial [1,2,3]. Graphene consists of individual layers of graphite, where graphite has shown fascinating characteristics and properties with a long history in many disciplines, particularly in engineering, physics, chemistry, and material science [4]. British Chemist B.C. Brodie and German scientist Schafhaeutl were the first to successfully isolate individual flakes of graphite through intercalation in mid-19th century [5,6]. In the late 1940s, P.R. Wallace suggested several extraordinary electronic and mechanical properties for individual graphite flakes through theoretical analysis [7]. Materials similar to graphene derived from graphite oxide were first described by Boehm et al. in 1962 in a diluted alkaline solution, with hydrogen sulphide, hydrazine, or iron salts utilized as the reducing agents [8]. Nearly 40 years later, in 2004, Geim and Novoselov used a micromechanical method and succeeded in producing extremely thin flakes of carbon from highly ordered pyrolytic graphite (HOPG), winning the Nobel prize in physics in 2010 [9,10]. A chronological arrangement of specific events in the history of synthesis, separation, and characterization of graphene is displayed in Figure 1 restructured from [4]. 

**Figure 2:**
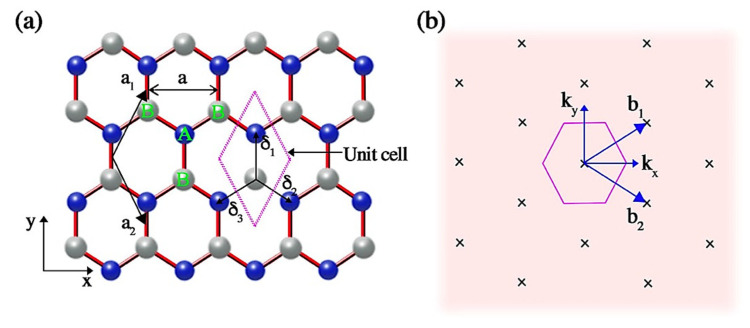
The honeycomb lattice of single-layer graphene, where gray and blue circles represent carbon atoms on (**a**) (B) sites and (**b**) the reciprocal lattice of single o layer graphene, where the shaded hexagon in light pink is the subsequent Brillouin zone.

Fundamentally, the graphene structure is an indefinitely extended two-dimensional (2D) sheet composed of sp^2^ hybridized carbon atoms organized in a hexagonal lattice [11] as illustrated in Figure 2. It is considered as one of the most useful allotropes amongst the entire family of nano-carbons because of its extraordinary characteristics, such as superior electron mobility (2.5 × 10^5^ cm^2^V^−1^s^−1^), excellent thermal conductivity (3000 WmK^−1^), good mechanical strength (Young’s modulus of 1 TPa), outstanding chemical stability, exceptionally high surface area (<2600 m^2^g^−1^), and excellent optical transparency (97.4% transmittance recorded at 550 nm) [12,13,14]. These characteristics can be finetuned further by altering different parameters such as defect density, porous structure, and number of layers. Thanks to these merits, graphene and a large variety of graphene-based nano-hybrids have been developed and used in a wide variety of real-world applications in various fields such as electronic [15], biomedical [16], sensors [17], energy storage [18], and environmental [19].

**Figure 3:**
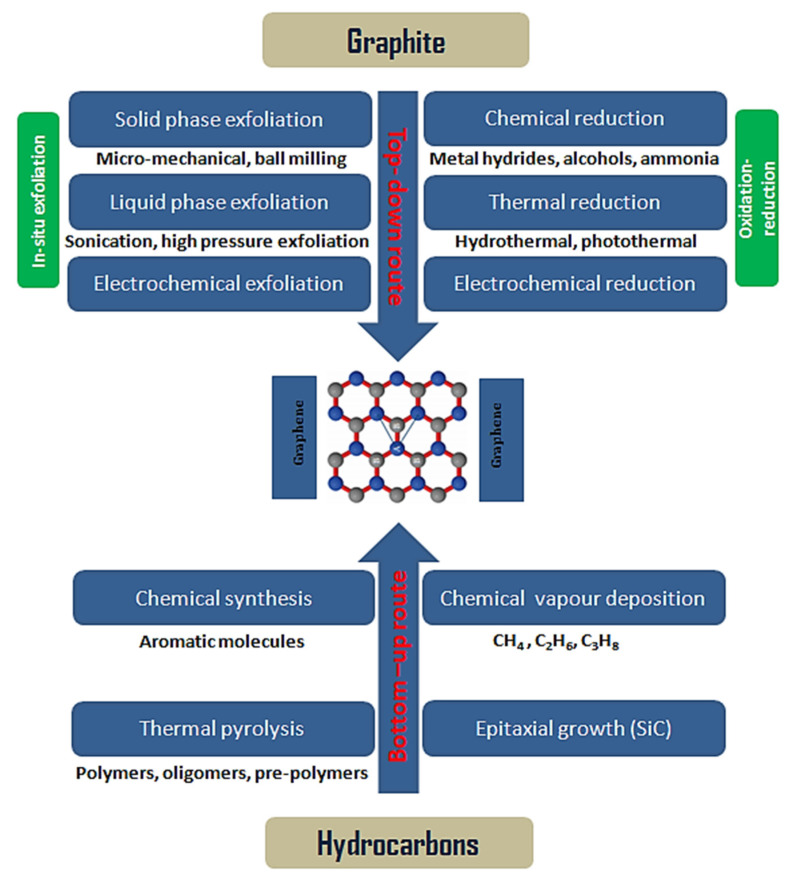
Schematic representation of most frequently used synthesis techniques of graphene.

Applications of graphene depend on numerous factors. Two key parameters are the number of layers and interlayer distance of produced graphene where layer numbers and separation distance can have a substantial impact on its properties and therefore its applications. π–π stocking and strong van der Waals attraction among layers of graphene can result in its layer aggregation. This impacts its properties negatively, e.g., a reduction in its available specific surface area, which results in a reduced number of active sites and sluggish ion diffusion kinetics for a significant number of applications including electrochemical energy storage, catalysis, water decontamination, gas sensing, and other environmental applications. Two main approaches are utilized to address these issues, which can result in improved specific surface area and enhanced accessibility of the porous structure. Firstly, chemical modification can improve the functionalities of graphene layers, and secondly, pore production in graphene or graphene layers and consequentially tuning its morphology can enhance the porous structure and improve graphene suitability for a broad range of applications. An application’s appropriateness regarding graphene also depends on the cost and quality of graphene and graphene-derived nanomaterials since these are the supplementary parameters defining its applicability in different applications and can vary subject to the desired application. For example, high-quality, defect-free graphene manufactured via chemical vapor deposition (CVD) is highly desirable for high-end electronics such as photodetectors, transistors, and large-scale transparent conducting electrodes [20]. Conversely, the use of graphene in environment-related areas including gas sensing or water purification requires a higher level of surface defects. This leads to an increase in its porosity and therefore enhances its chemically active sites, resulting in its enhanced performance [21]. Another key parameter in the production of graphene is its cost. The cost of graphene production depends on the techniques used. Graphene prepared through CVD is expensive and therefore this method is difficult to scale up for large-scale production. This leads to the synthesis of graphene by relatively cost-effective and more desirable methods such as thermal and chemical exfoliation of graphite. Since the cost of the most commonly used high-quality activated carbons has dropped from $150–200 kg^−1^ to $4–5 kg^−1^ over time, it would be impossible for graphene prepared via CVD to break into this segment of the market [22]. Therefore, most of the literature available on applications requiring larger graphene quantities, such as energy storage and environmental applications, are based on the production of graphene oxide (GO), reduced graphene oxide (rGO), chemically functionalized graphene, and graphene nano-powders using methods such as exfoliation. Materials produced using exfoliation or thermal growth methods are also competitive with highly cost-effective active materials such as activated carbon. Furthermore, graphene produced using these low-cost techniques consists of two or more than two-dimensional (2D) layers of graphene sheets stalked together to produce a three-dimensional (3D) structure, which possesses high defect density and high specific surface area. Therefore, the optimization of cost, quality, and properties of graphene according to its desired application is crucial for its large-scale production and commercialization. Figure 3 shows various synthesis techniques based on the fundamental principles of bottom-up and top-down processes to produce graphene. The most frequently used techniques for graphene synthesis will be discussed in detail in subsequent sections. Since this review will only cover the environmental applications of graphene, various associated parameters such as its porosity, chemical composition, physical structure, and surface chemistry will be discussed in detail as they are the key properties effecting its suitability for different environmental applications. A broad range of characterization techniques have been used extensively to examine the physical and chemical characteristics of graphene. It will be challenging to discuss these characterization techniques in detail here due to the size constraints and the scope of this review, where its focus is mainly related to graphene’s production approaches and its environmental applications. Nevertheless, some of the commonly used characterization techniques for graphene include X-ray diffraction (XRD), Raman spectroscopy, X-ray photoelectron microscopy (XPS), Fourier transform infrared (FTIR), and Brunauer–Emmett–Teller (BET). XRD is considered a useful non-destructive technique for the determination of interlayer spacing, the detection of impurities, and explaining structural strains [23,24], whereas Raman spectroscopy is a frequently used technique to provide valuable information regarding the number of layers, defects, and sp^2^ vibrations [25,26]. FTIR spectroscopy is another physiochemical technique used for the qualitative identification of a number of surface functional groups including oxygen and nitrogen [27]. XPS is a very precise chemical technique used for accurate elemental analysis to explore the chemical makeup of near-surface graphene-based samples [28]. The porous structure is usually examined by the BET method, which provides useful information on the surface area, pore size, pore size distribution, and pore volume [29]. 

**Figure 4:**
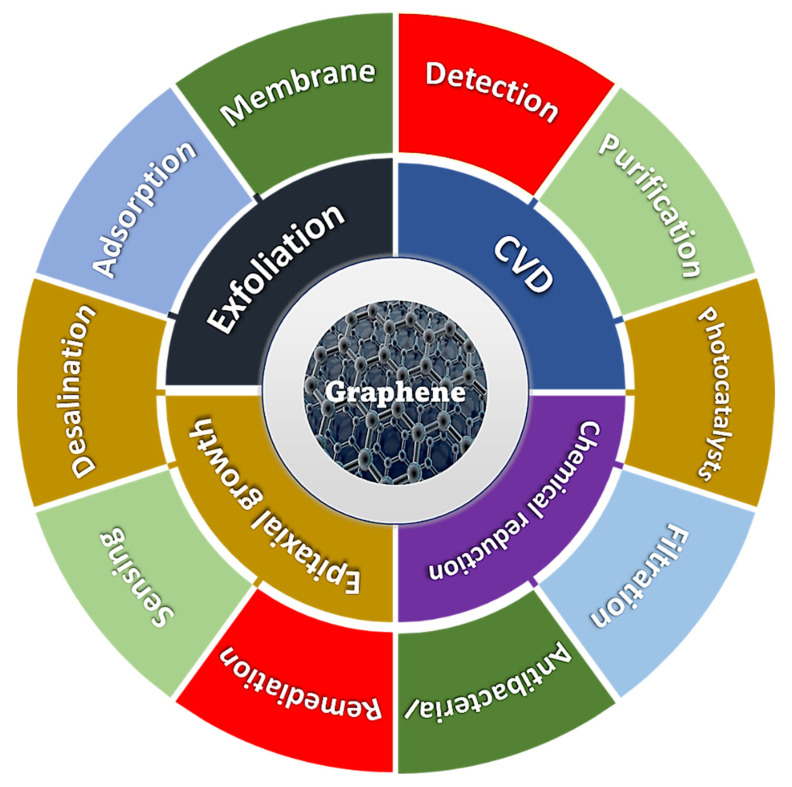
Graphical interpretation of synthesis techniques and environmental applications of graphene.

The rapid industrialization and modernization of transport infrastructure and the urbanization of countries have resulted in increased air pollution, with subsequent environmental impacts [30]. This occurs through the discharge of harmful compounds (gaseous, liquid, and solid) such as CO_2_ [31], CH_4_ [32] heavy metals [33], dyes [34], pesticides [35], and many other volatile organic compounds (VOCs) into the environment [33]. Release of these harmful pollutants not only damages the surrounding environment through water contamination, air pollution, global warming, and depletion of the ozone layer but also effects human health directly [21]. Therefore, the accurate detection and efficient elimination of these pollutants is mandatory to achieve environmentally friendly and sustainable growth. A diverse range of nanomaterials including carbons, metal organic frameworks, and conducting polymers have been utilized for a number of environmental applications to reverse the aforementioned trends [36,37,38,39]. Graphene, its derivatives, and hybrids are also being developed for adoption in a wide range of environmental applications including membranes for water treatment, high-porosity adsorbents for decontamination applications, and active materials for contamination-monitoring sensors [40]. Numerous research studies have been conducted on the synthesis and applications of graphene [41,42]. Literature is widely available on both bottom-up and top-down synthesis techniques of graphene [43]. Similarly, extensive literature is available on the applications of graphene in general and environmental applications in particular [3,44,45,46]. However, the availability of literature covering both the synthesis and environmental applications of graphene, its derivates, and hybrids is very limited. Therefore, this review will address both the production and environmental applications of graphene and will be distinctive from other previous studies in this area. The various synthesis methods and environmental applications of graphene are summarized in Figure 4.

This review also discusses, in detail, the advancements, advantages, and disadvantages of some of the most commonly adopted bottom-up synthesis techniques, including CVD and epitaxial growth, and also some of the top-down synthesis methods such as exfoliation (liquid, chemical, electrochemical, and mechanical) used for the synthesis of graphene. In addition, a number of environmental applications including water purification, gas sensing, metal ion sensing, and adsorption using graphene, GO, rGO, and graphene-based hybrids are comprehensively covered in this review. 

## 2. Graphene Synthesis

There are two key graphene synthesis routes, and they can be categorized as (i) top-down and (ii) bottom-up techniques and are schematically represented in Figure 3. In top-down methods, bulk material such as graphite is transformed into its smallest constituents to produce graphene [11]. Some of the major top-down techniques include [47] liquid-phase exfoliation [48], plasma etching [49], electrochemical exfoliation [50], laser ablation [51], ball milling [52], and chemical reduction [53]. Conversely, in the bottom-up synthesis route, graphene is produced through the decomposition of carbon-containing precursors (both gaseous and liquid) followed by the formation of a hexagonal structure of graphene layers [54]. Chemical vapor deposition (CVD) [55], thermal pyrolysis [56], and epitaxial growth [57] are some of the focal bottom-up approaches frequently used for the production of graphene. Frequently adopted production methods using both top-down and bottom-up strategies will be reviewed briefly in the sub-sections below. 

### 2.1. Chemical Exfoliation

Chemical exfoliation is considered an exceedingly efficient and cost-effective top-down synthesis process when compared with other widely used techniques such as epitaxial growth, micromechanical cleavage, and Hummer’s methods. In the chemical exfoliation method, graphene is produced from bulk graphite through exfoliation [58]. By selecting a suitable starting graphite material, the layer numbers and lateral size of the produced graphene can be controlled on a large scale through this process [59]. During chemical exfoliation, graphite layers are isolated using either reducing solvents or by oxidation. The main purpose of oxidation or reducing solvents is to reduce the van der Waals force by increasing the interlayer spacing between graphite flakes. Solvents such as hydrazine hydrate [60], N-methyl-2-pyrrolidone [61], and methanesulfonic acid [62] have been commonly used as reducing agents. 

**Figure 5:**
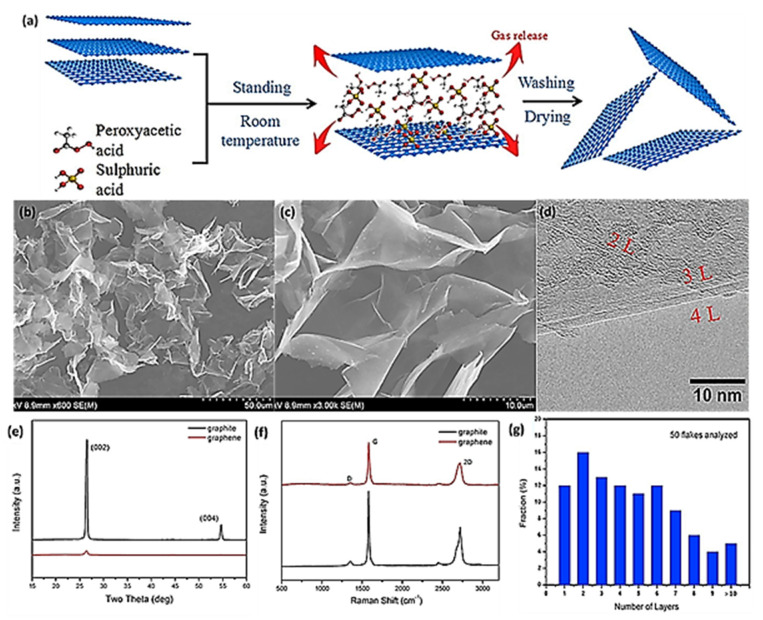
(**a**) Graphical representation of graphene production via chemical exfoliation; (**b**,**c**) SEM micrographs of as-obtained graphene; (**d**) HR-TEM image; (**e**) XRD spectra of graphite and pristine graphene; (**f**) Raman spectra of graphite and pristine graphene; and (**g**) thickness of graphene with diverse number of layers represented statistically. Adopted with permission from [65].

Superior-quality graphene with a minimum number of defects can be produced using this technique, and Gebreegziabher et al. recently reported the one-step synthesis of good-quality GO and rGO using chemical exfoliation, where reduced graphene oxide was prepared using H_2_SO_4_/KMnO_4_ assisted by ultrasonication. By controlling the reaction parameters, they managed to produce GO and rGO with a minimum number of defects [63]. Chemical exfoliation is a highly efficient synthesis technique; however, extremely hazardous oxidizing agents such as KMnO_4_ are traditionally used in this technique, which makes it less environmentally friendly. Attention has now been directed toward the use of less harmful chemicals as oxidation agents, and several studies have been conducted using chemical exfoliation with less harmful chemicals utilized as oxidization agents. In a recent study by Betancur et. al, nitric acid (HNO_3_) and sulphuric acid (H_2_SO_4_) were used to start the exfoliation of graphite, followed by the use of an ammonium hydroxide NH_4_OH solution to complete its exfoliation and partly reduce the resultant graphene samples [64]. In another study by Liu et. al, few-layer graphene (FLG) was prepared by the exfoliation of flake graphite using a binary system of peroxyacetic acid and sulfuric acid. This chemical exfoliation method stands out in terms of not only replacing hazardous oxidizing agents but also preventing the use of toxic reductants and elevated reduction temperatures [65]. Hazardous oxidizing agents and toxic reductants can result in harming the surrounding environment, whereas higher temperatures can lead to the structural damage of graphene and higher energy costs. This results in the production of inferior-quality graphene at higher costs. However, in Liu’s study, the swift production of good-quality few-layer graphene (FLG) was achieved using a novel binary-component system comprising peroxyacetic acid and sulfuric acid without sonication and the utilization of elevated temperatures, where graphene was produced at room temperature by chemical exfoliation in four hours [65]. The schematic of the entire production process is represented in Figure 5 where, (a), (b), and (c) display the highly delaminated morphological structure of graphene proving the high yield conversion of natural graphite into FLG sheets. XRD patterns are shown in (e) where the retention of both peaks at (002) and (004) confirms the preservation of the graphitic structure in graphene flakes, while (d) illustrates the presence of mono-layered, bi-layered, and tri-layered graphene sheets shown by HR-TEM image. Distinctly different 2D peaks for graphene and graphite around 2692 cm^−1^ are shown in (f), demonstrating the formation of FLG, and (g) displays the statistical analysis, revealing the number of graphene layers and average sheet thickness, which are observed to be less than five [65].

Chemical exfoliation has enormous potential for the synthesis of GO/rGO for wider commercial applications; however, the use of hazardous agents in graphene production and elevated production temperatures make this production process less economical for the large-scale production of graphene, when compared with other widely used techniques. Therefore, this method requires further investigation and research to make it a more viable technique both in terms of environmental friendliness and cost-effectiveness for large-scale production.

### 2.2. Mechanical Exfoliation

**Figure 6:**
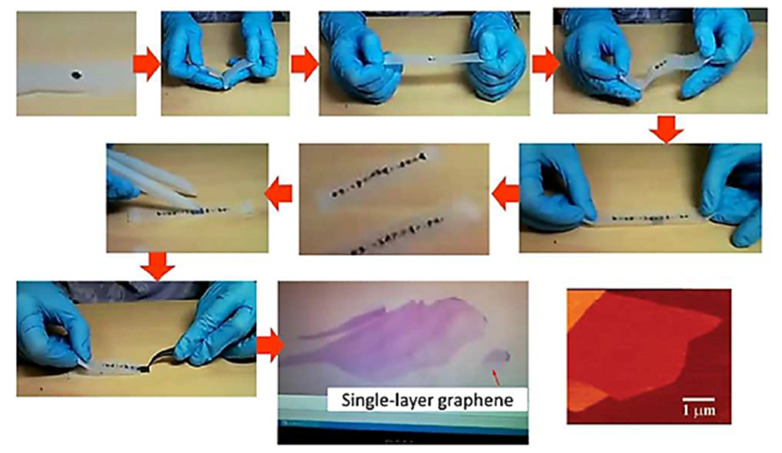
Illustration of ‘Scotch tape’ method to synthesize graphene. Adopted with permission from [67].

Mechanical exfoliation includes a number of top-down strategies such as micromechanical cleavage, ball milling, sonication, intercalation, and liquid-mediated exfoliations to produce graphene. The micromechanical cleavage method has been widely used to produce monolayers of the best-quality graphene with lateral dimensions in the µm scale. Novoselov et al. were the first to employ micromechanical cleavage, also known as the Scotch tape technique, to separate few-layer graphene flakes from a piece of HOPG in 2004, earning them the Nobel prize in physics in 2010 [9,66]. The different steps of graphene synthesis using this procedure are shown below in Figure 6.

Since graphite consists of monolayers of graphene closely stalked by weak van der Waals forces with an interlayer distance and bonding energy of 3.34 A and 2 eV/nm^2^, respectively [68], partially filled π orbitals perpendicular to plane sheets result in weak stacking of these graphene sheets involving van der Waals forces to form graphite and can break easily resulting in the production of excellent-quality graphene flakes. An external force of ~300 nNµm^−2^ is required to remove mono-layer graphene from graphite using mechanical cleaving [69]. The production of excellent-quality monolayer graphene is achievable with the deployment of the micromechanical cleavage method, which can reveal a large number of outstanding properties of graphene. However, this technique is exceedingly time consuming and laborious, limiting its use to mainly laboratory-based research, whereas achieving scalability using this method seems impossible. To address these issues, new strategies such as ball milling (dry and wet) and sonication have been developed, which are proving to be quite successful strategies for the large-scale production of graphene. 

**Figure 7:**
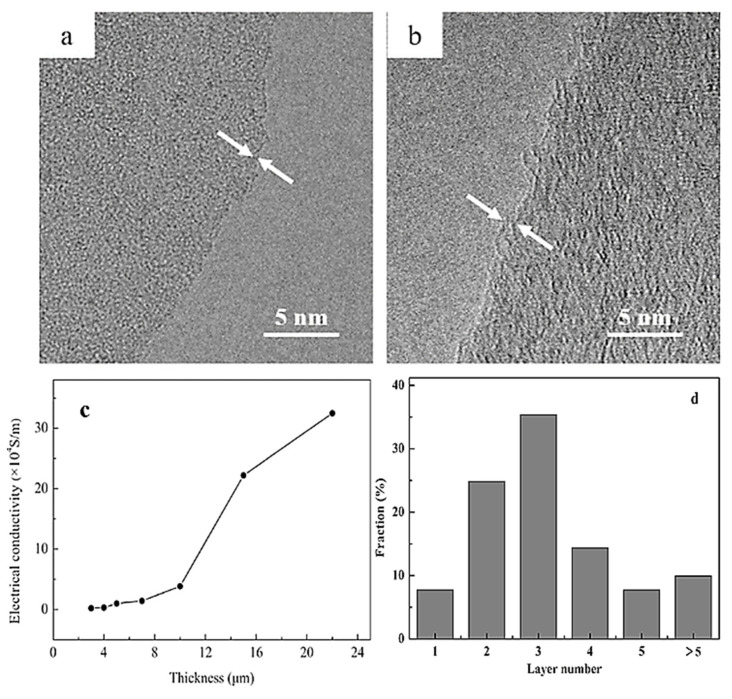
HR-TEM micrographs of (**a**) single-layer and (**b**) double-layer graphene; (**c**) electrical conductivity vs. sample thickness; and (**d**) presentation of graphene % in the form of histogram as a function of number of layers counted using AFM. Adopted with permission from [75].

Ball milling has been used as a substitute mechanical technique to synthesize good-quality graphene for industrial-scale production. In this process, shear force is applied laterally to exfoliate graphite into graphene flakes. There are two forces at play, i.e., lateral force and vertical force. Lateral force is highly desirable since it assists in the production of large-sized graphene flakes, whereas vertical force is less attractive since it results in reducing the flake sizes, or in some cases, even makes material completely amorphous. Therefore, it is always endeavored to keep the vertical force to a minimum in order to achieve good-quality, large-sized graphene flakes. Ball milling can be either dry or wet, where the latter can be further divided into two types known as planetary ball milling and stirred-media ball milling and are frequently deployed to produce graphene [70,71]. Wet ball milling uses a surfactant such as N,N-Dimethylformamide (DMF) or N-methyl-2-pyrrolidone (NMP)NMP, which have similar surface energy to that of adjacent graphene flakes and assist in disintegrating graphene layers from graphite. Moreover, dry ball milling is also employed widely to prepare graphene, by milling a mixture of chemically inert water-soluble inorganic salts and graphite followed by washing or sonication steps [72,73,74]. In a very recent study, ball milling in series with a shear-mixing exfoliation procedure was used with CO_2_ in supercritical condition to produce good-quality FLG, with more than 90% of the produced graphene having less than five layers. This method was found to be scalable, with each batch producing approximately 40 g of graphene, which is an exceptionally high quantity when compared with some of the other state-of-the-art techniques that are being applied presently. The prepared graphene possesses excellent properties such as high electrical conductivity of approximately 3.25 × 10^5^ S^−1^m on the PVDF substrate. Figure 7 illustrates various characteristics of prepared graphene where (a) and (b) show HR-TEM micrographs of single-layer and double-layer graphene, (c) shows variation in the electrical conductivity with the thickness of graphene samples, and (d) is the graphical representation of graphene % as a function of the number of layers counted using AFM [75].

**Figure 8:**
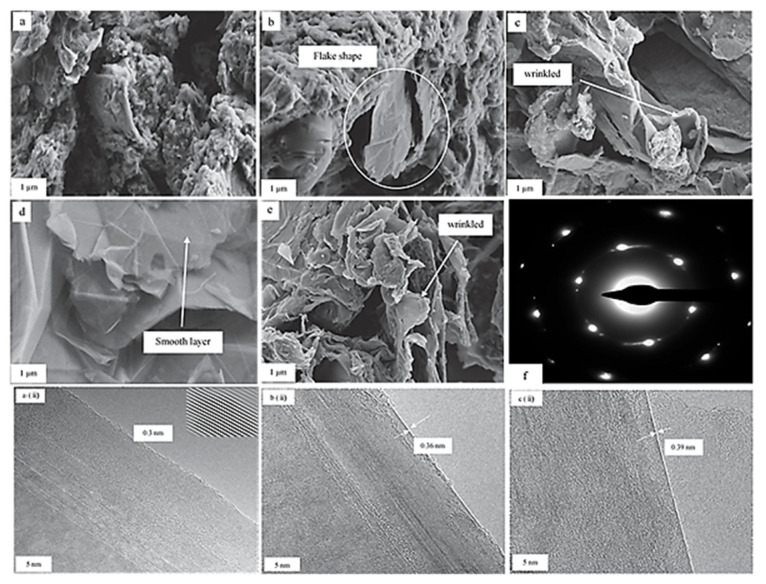
FESEM images: (**a**) Pure graphite; (**b**) graphene obtained after exfoliating in H_2_SO_4_ electrolytes without sonication time and with varied durations of sonication of (**c**) 15 min, (**d**) 45 min, and (**e**) 60 min. HRTEM micrographs of graphene produced using (**aii**) H_2_SO_4_, (**bii**) (NH4)_2_SO_4_, and (**cii**) H_3_PO_4_ electrolytes for sonication of 45 min at magnifications of 5 nm. (**f**) SAED pattern of graphene attained using H_2_SO_4_. Adopted with permission from [78].

Sonication-assisted liquid-phase exfoliation (LPE) is another mechanical technique where ultrasonication is utilized to extract individual layers of graphene after the successful exfoliation of graphite. Sonication-assisted LPE mainly involves three stages: (i) Graphite dispersion in specific solvent, (ii) sonication, and (iii) centrifugation [76]. This approach is exceptionally fascinating and opens a new area of research to produce graphene at a large scale very cost-effectively. However, one of the major drawbacks of this method is the extremely low concentrations of graphene suspensions, amounting to ~0.01 mgmL^−1^ in some cases, although different approaches, such as extended sonication time, have recently been trialed to increase graphene concentration [66]. A study by Lotya et al. showed that extending the sonication time results in a higher concentration, where an increased concentration of approximately 1.2 mgmL^−1^ was achieved after an exceptionally long sonication time of 460 h, which resulted in yields of up to 4%wt of monolayers [77]. Different sonication times and solvents are also being used to prepare graphene with superior properties. A detailed study Htwe et al. used three different solvents and sonication times to optimize various characteristics of produced graphene. They observed that using a sonication time of 45 min and H_2_SO_4_ as a solvent produces graphene with the smallest crystallite size, excellent electrical conductivity, the smaller number of defects, and the least interlayer distances [78]. Figure 8a–e represent FESEM micrographs of pure graphite and graphene at a magnification of 1 µm. Figure 8a characterizes pure graphite, (b) represents graphene produced without sonication, whereas (c), (d), and (e) represent graphene produced using H_2_SO_4_ at sonication times of 15, 45, and 60 min, respectively, and it is evident that graphene produced in H_2_SO_4_ with a sonication time of 45 min is of the best quality, with smooth graphene flakes without any wrinkles. Figure 8a(ii),b(ii),c(ii) are HRTEM micrographs of graphene produced using different solvents, displaying the interlayer distances. It is evident that graphene produced using H_2_SO_4_ as the solvent has the lowest interlayer distance of 0.3 nm. Figure 8f shows the selected area electron diffraction (SAED) pattern of graphene prepared when using H_2_SO_4_ as the solvent, exhibiting six-fold symmetric diffraction with the hexagonal crystal structure for graphene exfoliated using H_2_SO_4_ as an electrolyte, which is the typical crystal structure of graphene [78].

Mechanical exfoliation can be a useful technique for the mass production of graphene; however, this method has its own weaknesses, e.g., it is a low-yield technique, and the production of monolayers is especially very low when mechanical exfoliation is used. Even though a limited number of studies, as discussed above, with higher yield have been successfully conducted when using this technique, these works have been performed at the laboratory scale only. Higher yields are only possible when very long exfoliation times are used, and this is unsustainable both in terms of time and for commercial applications. Furthermore, all mechanical exfoliation techniques suffer from undesirable fragmentation effects resulting in smaller-sized graphene flakes. Mechanical exfoliation can also result in a large amount of unexfoliated graphite requiring centrifugation as an extra step. Reproducibility using mechanical exfoliation is another production issue and requires extra attention. Therefore, graphene production through mechanical exfoliation yet requires an immense amount of research and development (R&D) work to make this technique a commercial success for large-scale development. 

### 2.3. Electrochemical Exfoliation

**Figure 9:**
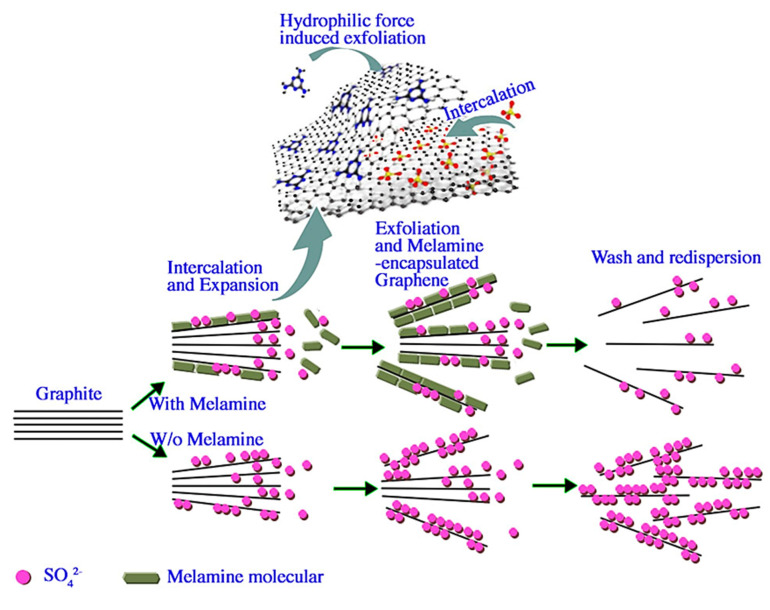
Graphical representation of electrochemical exfoliation process with/without melamine additives. Reconstructed with permission from [81].

Most recently, the production of graphene through electrochemical exfoliation has turned out to be an effective top-down technique. Advantages of electrochemical exfoliation over other synthesis strategies include a short synthesis duration, a facile production process, low instrumental cost, and the possibility of production and modification of good-quality graphene [50,79]. During this process, fixed voltages are applied to graphite electrodes, which leads to the weakening of van der Waals forces (5.9 kJ mol^−1^) among graphite layers resulting in the separation of layers [80]. A schematic of the electrochemical exfoliation of graphene is presented in Figure 9 [81]. Graphene layers are accumulated on graphite electrodes according to the applied potential, which provides the foundation for the categorization of exfoliation known as anodic (applying a positive bias) type and cathodic (applying a negative bias) type exfoliation. 

**Figure 10:**
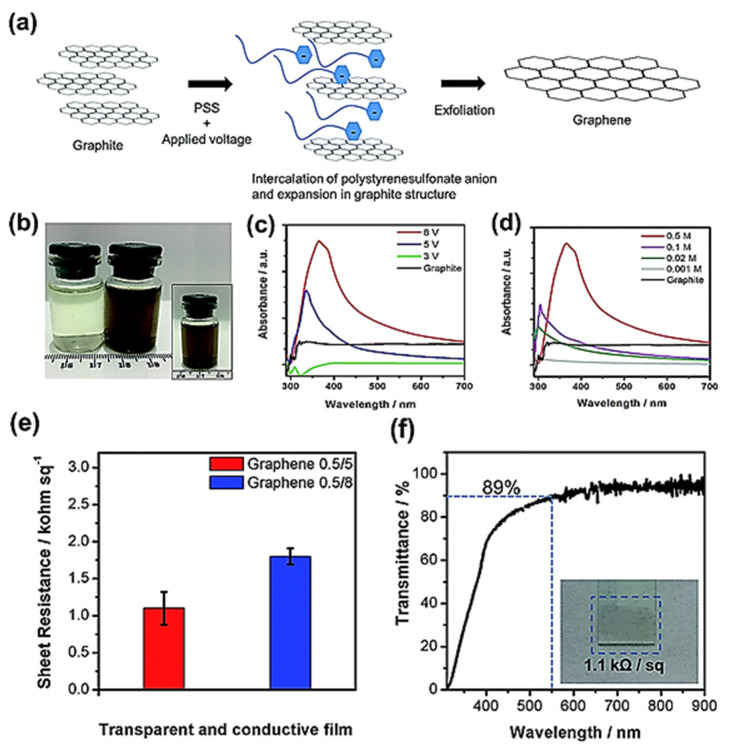
(**a**) Schematic of graphene production process, (**b**) 0.5 M PSS solution graphene-PSS dispersion acquired under 8V DC. (**c**,**d**) The UV-vis profile at varying and 8V DC voltage, respectively. (**e**,**f**) Transmittance and sheet resistance-produced samples. Reproduced with permission from [81].

This method can also provide a cleaner, greener, and environmentally friendlier route to produce good-quality graphene from recycled graphite such as electrodes of spent batteries. Prakoso and co-workers used rods of graphite of spent Zn–C batteries and produced excellent-quality graphene with high transmittance of approximately 89% and low sheet resistance of 1.1 kΩ sq^−1^, and these characteristics of prepared graphene are analogous to those obtained by more complicated and energy-intensive techniques such as CVD. The complete synthesis procedure and various characteristics of the graphene produced by this technique are presented in the Figure 10 [81]. Figure 10a displays the schematic of the graphene production process, Figure 10b shows a 0.5 M PSS solution, whereas Figure 10c,d show the UV-vis spectra at varying and 8V DC voltage, respectively. Figure 10e,f show the sheet resistance and transmittance of the produced graphene. 

Traditionally, graphite exfoliation is performed in an aqueous solution [82], and there are three key parts of the exfoliation process when aqueous solutions are used [83]:

1. Water electrolysis and the production of oxygen and hydroxyl radicals.
2. The movement of hydroxyl and oxygen radicals and opening of graphite edges.
3. Intercalation of the species of electrolyte and, subsequently, gas formation for the expansion of graphite.

Different studies have also been performed using other solutions such as organic electrolytes, e.g., recently, Swapan and co-workers successfully produced multilayer graphene using a pure organic solution of tetramethyleammonium hydroxide (TMAH; (CH_3_)_4_NOH) dissolved in water as an electrolyte, where various distinctive properties of FLG were characterized using a range of methods including Raman spectroscopy, FTIR, XRD, AFM, and UV-Vis analysis, confirming the production of good-quality graphene [84]. As with any other technique, electrochemical exfoliation has its advantages and disadvantages. This process can be fast, efficient, scalable, and environmentally friendly and can result in producing good-quality graphene with a high yield and excellent electronic properties; however, this method can suffer from an inhomogeneous thickness and slightly uncontrolled oxidation of graphene flakes, which can occur during the synthesis process of graphene layers, which are undesirable properties for many applications.

### 2.4. Liquid-Phase Exfoliation

**Figure 11:**
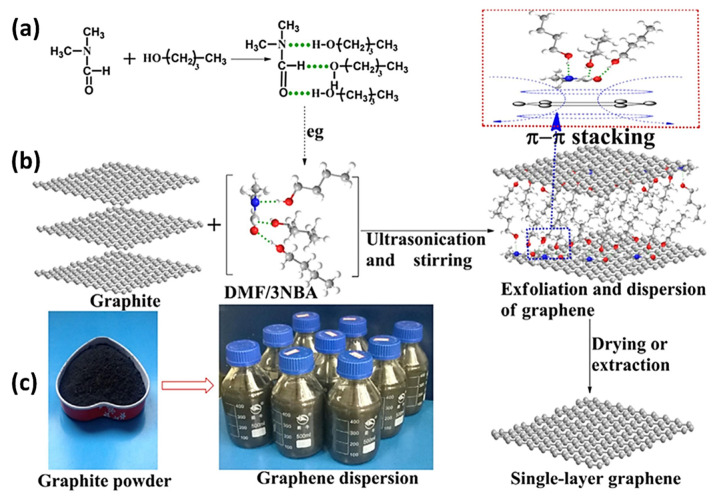
(**a**) Hydrogen bond formation between dimethylformamide (DMF) and n-butanol (NBA) molecules; (**b**) schematic of the graphite exfoliation into few-layer graphene/single-layer graphene; (**c**) powder of graphite and dispersion of graphene-DMF/3NBA. Adopted with permission from [87].

Different synthesis routes have different strengths and shortcomings, and bottom-up techniques such as CVD and epitaxial growth can be advantageous to synthesize high-quality graphene with a controlled number of layers and large sizes of graphene flakes. However, these methods are inappropriate for industrial-scale production owing to their energy-intensive nature and restricted dimensions. Conversely, top-down techniques, such as liquid-phase exfoliation, can be used for the commercial production of graphene thanks to their simplicity and scalability, where mono-layer or FLG can be produced by exfoliation of natural graphite by high shear mixing or sonication [85]. Liquid-phase exfoliation has seen immense progress after its first successful use via the sonication of graphite powder in N-methylpyrrolidone (NMP) by Hernandez et al. in 2008 [86]. Sonication-assisted liquid-phase exfoliation has been widely used for graphene synthesis, where the size of graphene flakes and distribution can be controlled. A recent study in which sonication-assisted liquid-phase exfoliation was used for graphene synthesis demonstrated that ultrasonic waves can be employed to control the size and thickness distribution of graphene sheets. Figure 11 shows the complete process of graphene synthesis. The proposed hydrogen bonding between various DMF and NBA molecules shown in Figure 11a,b represents the exfoliation of graphite into FLG and SLG schematically, and Figure 11c shows the graphite powder used and graphene-DMF/3NBA dispersions [87]. 

**Figure 12:**
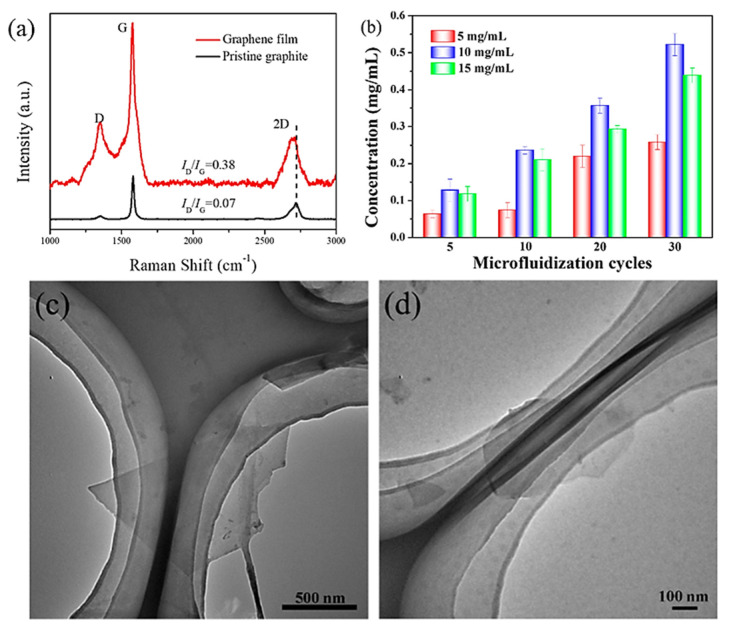
(**a**) Raman spectra of as-prepared graphene and pristine graphite; (**b**) concentration of graphene with the number of microfluidization cycles; and (**c**,**d**) TEM micrographs after 20 and 30 microfluidization cycles, respectively. Adopted with permission from [88].

Sonication-assisted liquid-phase exfoliation has its own constraints since it has low efficiency and uses high-energy-consumption processes [32]. This has resulted in the development and use of alternative liquid-phase exfoliation approaches such as microfluidizer methods, which use high shear mixing, which is a facile method for graphene production at ambient pressure and temperature and has immense potential for the production of good-quality graphene at a large scale through the effective exfoliation of graphite [11,88]. In a recent study, successful graphite exfoliation for the large-scale synthesis of excellent graphene using sodium salt of a styrene-maleic anhydride copolymer (SMA) as a stabilizer and utilizing the microfluidizer method was used to produce few-layer graphene. High concentrations of 0.522 mgmL^−1^ of graphene with high-quality graphene consisted of the production of uniform flakes of less than 1µm. Thermal conductivity and tensile strength were superior for the produced graphene by ~28.8% and ~32.6%, respectively, using this procedure when compared with the subsequent values determined for pure PA66. Figure 12a shows the Raman spectra of as-prepared and pristine graphene, where the low I_D_/I_G_ ratio of 0.07 signifies the excellent quality of graphene, and Figure 12b shows the graphene concentration vs. the number of microfluidization cycles with the highest concentration of approximately 0.522 mgmL^−1^ obtained after 30 cycles. Finally, Figure 12c,d show the transmission electron microscopy (TEM) micrographs representing the production of FLG and SLG after 20 and 30 cycles, respectively [88]. 

As discussed above, liquid-phase exfoliation has the potential to be used for the large-scale preparation of good-quality graphene since it is a simple, scalable, and environmentally benign technique. However, this process has its own downsides, for instance, its low production of mono layers, inhomogeneous distribution of graphene flakes, and highly energy intensive, costly, and time-consuming characteristics. Keeping in mind the potential of this technique, it is anticipated that it requires an immense amount of work to become a technically and financially viable process for graphene synthesis on a wider scale. 

### 2.5. Epitaxial Growth

Epitaxial growth of graphene is achieved through surface depletion of substrates such as silicon carbide (SiC). Since silicon has higher vapor pressure than carbon atoms, during high-temperature annealing of the SiC substrate, silicon desorbs first from the substrate surface, leaving a carbon-rich surface behind, followed by the growth of graphene [89,90], where fabricated layers of graphene using this technique are called epitaxial layers. The preparation of graphene using epitaxial growth on SiC is a promising approach to produce good-quality graphene with a larger area and uniform thickness. The production of highly ordered and clean epitaxial graphene films can be credited to the very high annealing temperature (<1400 °C) and high Ar pressure. An additional benefit of this technique over other bottom-up techniques such as CVD is the non-existence of the requirement to transfer graphene to other substrates, providing the ability to produce, with ease, electronic devices such as radio frequency (RF) transistors, field effect transistors (FET), integrated circuits (IC), and sensors directly on semiconducting or semi-insulating SiC [91,92,93]. Graphene synthesis on the SiC substrate (6H- and 4H-SiC) has been frequently used; however, achieving larger graphene areas with consistent thickness in a controlled manner continues to be an immense challenge. To address this issue, various other substrates such as ruthenium (Ru) have been used, and single-crystal graphene with dimensions exceeding 200 µm has been produced [94] using Ru as a substrate. 

**Figure 13:**
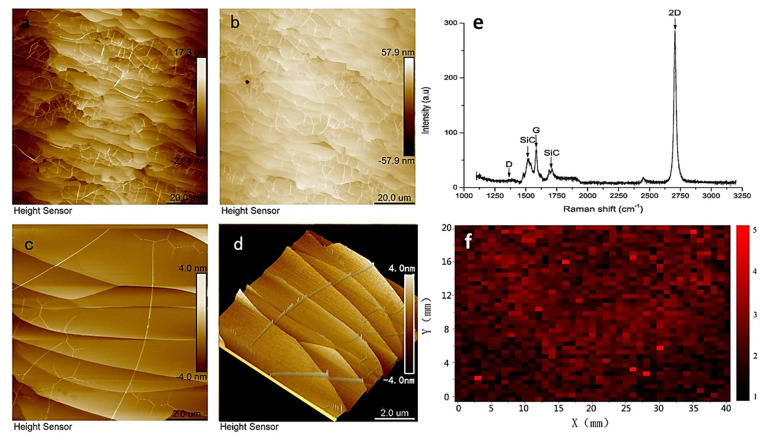
Topography of graphene layers using Atomic force microscope (**a**,**b**) in 100 × 100 μm^2^ random area. (**c**) AFM image of larger size (10 × 10 μm^2^) of a 4H-SiC graphene film. (**d**) 3D profile of epitaxial graphene in 10 × 10 μm. (**e**) Raman profile of graphene on SiC (0 0 0 1) substrate. (**f**) Raman mapping of graphene (I_2D_/I_G_). Adopted with permission from [95].

Although epitaxial growth is considered one of the best methods to produce large-area graphene, the application of an ultra-high vacuum (UHV) makes it extremely challenging to control the thickness of the layers of graphene. Moreover, the high sublimation rate of Si atoms results in creating a large number of defects, especially on C-face of the SiC substrtate, ultimately filling it with carbon. To address the issue of UHV and uncontrolled growth of the graphene layer, Zhao et al. recently adopted a new synthesis strategy to produce good-quality, large-sized graphene under a low-vacuum environment [95]. This method not only reduces the production cost, but also controls the growth rate of graphene on the SiC substrate. Several characteristics of graphene prepared by this method are shown in Figure 13. Atomic force microscope (AFM) images in Figure 13a,b show the formation of a continuous layer of graphene. Figure 13c,d display surface topographies in detail in 2D and 3D, measured at the same spot as AFM images. It is evident from Figure 13c that terraces of epitaxial graphene are exceptionally consistent and homogeneous, whereas Figure 13d shows the formation of nanometer-scale steps on the entire layer of graphene. Figure 13e shows typical Raman spectra of graphene produced through epitaxial growth on the SiC substrate, and finally, Figure 13f illustrates the Raman mapping to confirm the uniformity of graphene layers, where the ratio of 2D and G bands (I_2D_/I_G_) is greater than two in the entire region and is in line with the ratio of monolayer graphene [95].

Even though epitaxial growth has been successfully used to produce excellent-quality graphene with control over the number of layers and flake sizes, this production procedure is highly energy-intensive and difficult to control, particularly at elevated temperatures and Ar pressures, which can be a safety concern. Therefore, this technique requires more R&D work to bring it in line with other techniques. 

### 2.6. Chemical Vapor Deposition (CVD)

**Figure 14:**
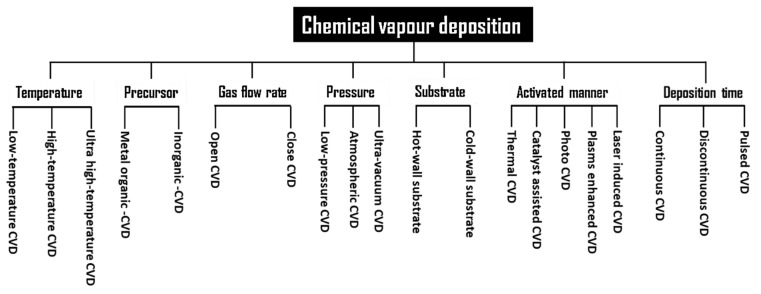
Classification of CVD methods. Reproduced with permission from [96].

CVD is a bottom-up technique and is by far the most adopted procedure to prepare good-quality graphene, with potentially large quantities of monolayer and few-layer graphene. In this method, graphene can be s synthesized by either the deposition of vapors from carbon containing gases such as CH_4_ and H_2_ on metal/dielectric surfaces or through the surface separation of carbon from metal/carbon solutions. There are a number of factors such as the reactor configuration, gas feedstock, gas ratios, partial pressure of gasses, temperature, growth time, and reactor pressure, which govern the type of processes and chemical reactions taking place inside a CVD reactor [96]. CVD deposition was first reported in 1966 where a crystalline graphite film was thermally deposited on a Ni substrate; this was followed by single-layer deposition of graphite by the CVD technique, where the Pt surface was used as the substrate and hydrocarbon decomposition as the source [97,98]. This technique has seen huge interest after the separation of single-layer graphene in 2004, which resulted in wider production and applications of graphene and graphene-based composites. A large number of chemical CVD methods are being developed and used. These synthesis methods are governed by characteristics of seven main preparation parameters, namely, the nature of the precursor, temperature, pressure, mix of gases, type of substrate, deposition time, and gas flow rate [99,100], which are shown in Figure 14 [96].

**Figure 15:**
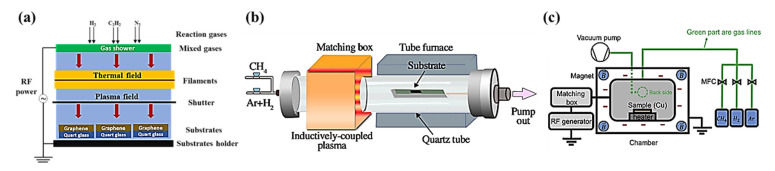
A graphical presentation of the RF-PECVD setup complemented with (**a**) HF, (**b**) ICP, and (**c**) CCP. Recreated with permission from [100,101,102,103].

All these methods have their advantages and disadvantages, but it is outside the scope of this paper to cover all these CVD techniques here. One of the effective CVD methods used for the synthesis of graphene is radio frequency plasma-enhanced CVD. Figure 15 shows the schematic of the radio frequency plasma-enhanced CVD (RF-PECVD) setup accompanied by different arrangements, i.e., hot filament (HF), inductively coupled plasma (ICP), and capacitively coupled plasma (CCP) [100,101,102,103].

The benefit of combining radio frequency with hot filament results in avoiding the annealing step, while graphene is directly deposited on a substrate such as Ni [104] as shown in **(a)**, whereas inductively coupled plasma systems as shown in **(b)** can be coupled with an inductive circuit element, which results in high growth rates of graphene [105]. Finally, **(c)** shows the capacitively coupled plasma, which is a simpler setup when compared with other techniques such as ICP, while it is also less energy-intensive; however, this can result in the formation of amorphous layers [106]. 

The main shortcomings of this process include toxic gaseous byproducts and the necessity of high operating temperatures (800–1100 °C). These high operating temperatures can be reduced significantly, and reduced temperatures of approximately 550–600 °C have been reported to synthesize graphene with the aid of plasma [107,108]. Furthermore, the transfer of prepared graphene from the used substrate, i.e., Cu, is a huge challenge since this can result in the introduction of surface defects. Researchers have developed various techniques to prevent the occurrence of these defects through the application of polymer films. For instance, a recently developed transfer procedure and adhesive have been used between the directed substrate and the graphene on Cu, which can be removed through etching after the successful transfer of graphene [109]. Successful studies have also been performed to produce transfer-free and defect-free graphene, where graphene has been produced on top of glass and PET substrates directly at very low temperatures (150 °C) and used directly for applications such as flexible electronics [110]. As discussed above, low-temperature growth of graphene has been achieved using CVD; however, this has been performed in very few research studies and requires more work to make it universal. Moreover, several challenges remain to be addressed, such as the quality of graphene where non-uniform and discontinuous deposition of graphene persists, and require further research work to explore the growth phenomenon of graphene at low temperatures in depth using the CVD method. 

Advantages and disadvantages including cast, scalability, applicability, environmental concerns, production time scale, quality of produced graphene, and production yields of different high-end synthesis methods for graphene are presented in Table 1.

Table 1 summarizes the various benefits and downsides of different synthesis approaches. For instance, mechanical exfoliation is advantageous in numerous aspects such as large-scale production and cost-effectiveness. However, several issues remained to be addressed including uncontrolled defects, the fragmentation effect, the random number of layers, flake sizes of produced graphene, and low yield, especially of monolayer graphene, when the mechanical exfoliation route is employed. CVD on the other hand can address some of these issues including the preparation of excellent-quality graphene and larger sizes; nevertheless, this method has its own drawbacks since it is extremely complicated and expensive. Therefore, cost-effectively producing superior-quality graphene with a precise structure in an environmentally friendly manner using a single synthesis approach is still an immense challenge. The number of graphene layers and achievable dimensions for graphene produced by different synthesis methods are also summarized in Table 2, where the mobilities listed in the table are for graphene transferred to Si/SiO_2_ wafers [115].

### 2.7. Trend Analysis of Different Graphene Synthesis Techniques

**Figure 16:**
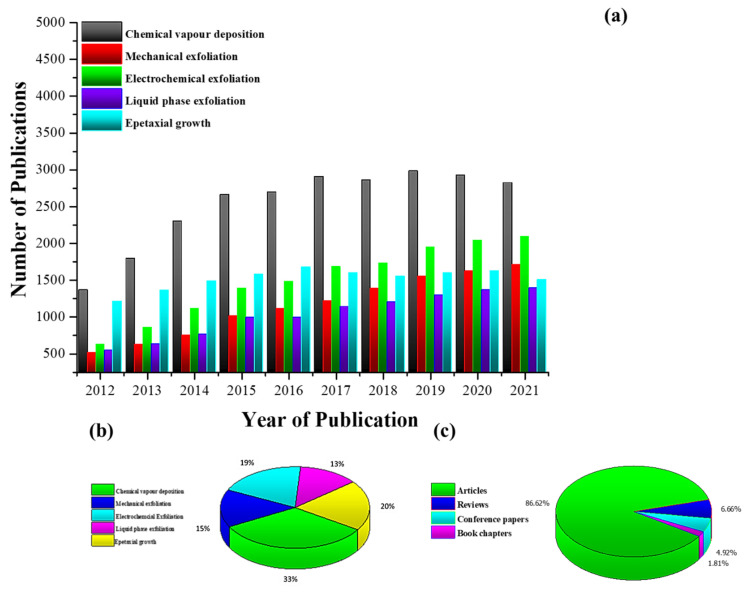
(**a**) Publication trend of graphene synthesis techniques from 2010 to 2021, (**b**) Percentage publications for each graphene synthesis techniques, (**c**) Type of publications of the graphene synthesis techniques. All self-extracted from Scopus (www.scopus.com) (accessed on 11 September 2022).

Graphene was isolated from natural graphite in the early 2000s by mechanical means; however, improvements in production processes over the past two decades have resulted in the development of a wide range of fabrication approaches to produce graphene. Each of these techniques have their own advantages and shortcomings influencing their applications. The authors extracted data from the past ten years from Scopus from a number of different types of publications, specifically research articles, review articles, conference papers, and book chapters on key synthesis techniques, as shown in Figure 16**,** to understand the past trends and future outlook of synthesis strategies of graphene. 

As can be observed from Figure 16a,b, CVD is by far the most researched and used technique when compared with other techniques, since this bottom-up method can result in the production of high-quality (low defects), large-sized graphene with a controlled number of layers. CVD has seen sustained growth up until 2019, with aa slight decrease in both 2020 and 2021, which may be due to the necessity of finding alternative techniques that are green and more sustainable. Alternative top-down techniques such as liquid phase and electrochemical exfoliation have seen steady growth over recent years since, by using electrochemical exfoliation, good-quality graphene can be produced using a simpler and more eco-friendly synthesis procedure at a much lower cost; however, this technique still faces various challenges including slight oxidation and non-uniform thickness of produced graphene. Similarly, liquid-phase exfoliation is another very promising technique, which has seen increased use recently and can be used to cost-effectively produce graphene at a larger scale; however, this top-down technique is time consuming and produces predominantly few-layer graphene. It can be seen from Figure 16c that most of these research publications are comprised of experimental articles, making graphene synthesis one of the most studied, active, and high-growth area of research.

Continuous growth in the number of publications, with more than one-fifth (21%) of these publications in open-access journals, gives an indication of the strong interest in research work in this field. It is anticipated that this trend will continue for years to come, since various characteristics of graphene, its derivatives, and graphene-based nanomaterials are still being uncovered, resulting in broadening of their application base. In addition, it is presumed that future research will mainly be focused on the cost-effective synthesis of graphene in an environmentally friendly manner to make graphene more desirable for commercial applications. This will shift the synthesis of graphene away from more commonly used traditional methods such as CVD toward improvements in more green and sustainable techniques currently being used and the development of new production processes. 

## 3. Applications of Graphene

Since its discovery, graphene has seen an overwhelming response from scientists working in diverse research areas such as engineering, energy storage/management, medicine, electronics, material science, and many other disciplines. Graphene, graphene oxide, reduced graphene oxides, and its composites have been widely adopted as active materials in a wide range of applications including electrochemical energy-storage devices (EESDs) such as supercapacitors and electrochemical batteries [116,117,118]. Thanks to their superior characteristics such as excellent thermal, electrical, mechanical, and optical properties, graphene-based materials have also been widely used in electronic applications. Furthermore, graphene and its derivatives have also seen enormous applicability in optical devices including photodetectors, electronic sensors, light-emitting diodes (LEDs), and other applications including temperature sensors, transducers, thermoelectric sensors, and energy-management systems [119,120,121].

Graphene’s derivatives have evolved and diversified over the past two decades and have resulted in immense progress in their processing and applications. In particular, they have seen recent applications in biomedicine due to their versatility in the synthesis of quantum dots, nanosheets, and nanoparticles where their novel electrical, thermal, optical, mechanical, and magnetic properties make them superior materials for biomedicine applications. The use of graphene in biomedicine includes, but is not limited to, thermal biosensors, biomolecule sensors, drug delivery, tissue engineering, bioimaging, and photothermal and photodynamic therapies [122,123,124,125].

Lastly, graphene and it’s derivates and nanocomposites have also been widely used in a wide range of environmental applications such as membranes for the detection and removal of contaminants from wastewater, active materials for gas sensors, carbon storage and conversion, water desalination, electrocatalysis, photocatalysis, and agricultural pollution sensors [126,127,128,129,130]. It is a daunting task to cover the entire array of applications of graphene and graphene-based composites and hybrid materials in a single article. However, in this article, a number of leading environmental applications of graphene will be discussed in detail.

### 3.1. Gas Sensing Applications

Gas sensors work on the basic principle of converting gas volume fractions into corresponding electrical signals [131]. Gas sensors are considered particularly important technology to detect and quantify various hazardous and toxic gases in a number of fields including the manufacturing industry, medicine, agriculture, and in wider/diverse environment settings. Improvements in sensor technology require advancements in various performance characteristics such as the response time, selectivity, power consumption, stability, repeatability, and sensitivity, which can be achieved by exploring and deploying new and state-of-the-art sensing materials [132]. In the last few decades, a broad range of nanomaterials have seen increased interest in the field of gas sensing. Two key motives behind the development of new sensing materials are improving the surface activities and lowering the effective operational temperatures of gas sensors. Since most of the gas sensors need comparatively higher working temperatures to have enhanced gas sensing responses, these higher operating temperatures give rise to issues such as increased energy costs and challenges of thermal management [133]. Whereas, to improve surface activities, nanomaterials with high specific surface area and rich surface chemistry are more desirable, thereby improving devices’ sensitivity and sensing kinetics. Among other nanomaterials, 2D layered nanomaterials including graphitic carbon nitride, metal dichalcogenides, and graphene have received enormous attention thanks to their extraordinary thickness-dependent physical, chemical, and electrochemical characteristics, including high surface-to-volume ratios and strong surface activities, which result in excellent sensitivity attributable to very strong interactions between gas molecules on their surface [134]. Strong molecular interactions on the surface of these materials not only improve sensors’ performance but this performance enhancement can also be attained at relatively low operational temperatures. Among other 2D materials used in in sensor applications, graphene is the most researched material due to its diversity of synthesis routes and its extraordinary properties such as ultra-high surface area, chemical inertness, and exceptionally high charge carrier mobility [135]. It has been used in gas sensing applications in various forms such as pristine graphene (PG), graphene oxide (GO), reduced graphene oxide (rGO), and graphene hybrids.

**Figure 17:**
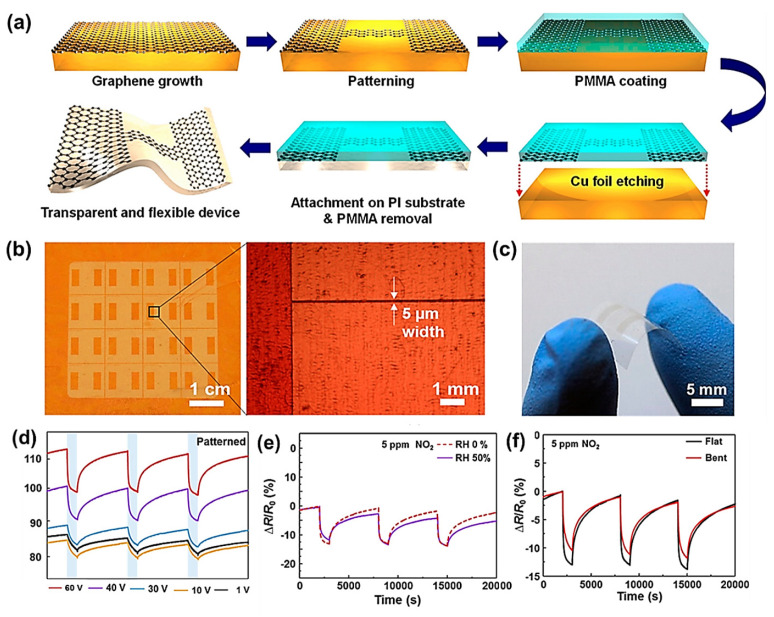
(**a**) Production process of PG sensor, (**b**) optical micrographs of patterned graphene on top of copper foil, (**c**) photo of a fabricated PG gas sensor on a PI substrate, (**d**) response curves under different bias voltages, (**e**) output under relative humidity conditions of 0% and 50% at 60V, and (**f**) response without and with the bending strains. Adopted with permission from [140].

PG is a carbon nanomaterial of single-atom thickness with a 2D structure possessing outstanding properties, making it highly desirable for gas-sensing applications when compared with other 2D materials, especially for the detection and quantification of very low concentrations of gasses, since the surface of PG is highly sensitive, where every single atom is available on a single layer of graphene for adsorption and desorption processes to occur [136]. As discussed earlier, among other synthesis processes, CVD is the preferred technique to produce superior-quality large-size PG sheets. During this process, hydrocarbons are catalytically decomposed and deposited on typical metal substrates including cobalt, nickel, and copper followed by the transfer of these graphene sheets to arbitrary substrates (flexible or nonflexible) to produce different devices including gas sensors [137,138]. Kim et al. used PG sheets prepared by CVD to manufacture a transparent self-activated gas sensor and investigated its endurance in mechanical bending, diffident levels of humidity, and applied voltages. A schematic of the fabrication procedure of graphene sensors is shown in Figure 17a. Self-activation of gas sensors was realized by inducing current crowding in patterned narrow electrical channels of three-layer graphene using a flexible and transparent substrate as shown in Figure 17b,c, where the width and length of the channels were maintained at approximately 5 μm and 5 nm, respectively, for the purpose of reproducibility. This flexible sensor made of graphene sheets was investigated under different operational conditions, where increasing the bias voltage resulted in their enhanced response and recovery as shown in Figure 17d. The insignificant impact of humidity on this self-activated sensor was observed, as shown in Figure 17e. Furthermore, thanks to the excellent flexibility of graphene, the sensor displayed consistent operation under mechanical bending (Figure 17f) [139,140].

In certain circumstances, the gas detection ability of graphene is significantly reduced, i.e., in case of NH_3_ where weak bonding (~20 meV) and inferior charge transfer (~0.027 e) exist between graphene and NH_3_ [141]. To reduce these limitations and improve sensors’ performance, intentional functionalization has been proposed and effectively utilized. However, functionalization of graphene normally performed with the application of chemical processes can damage its fundamental electrical properties through surface covalent bonding. Furthermore, these chemical modifications commonly require very complex procedures and harsh reaction conditions, necessitating modification in synthesis processes by employing environmentally friendly and facile synthesis procedures for the preparation of functionalized graphene [142]. In a recent study by Huang et al., the liquid-phase exfoliation route was adopted to prepare decent-quality functionalized graphene with very few defects using the non-toxic and widely available compound flavin mononucleotide sodium (FMNS), which is a derivative of B_2_ as an efficient stabilizer. It was observed that FMNS-derived graphene (G-FMNS) showed excellent sensitivity toward NH_3_ where FMNS not only provided the perfect active sites for ammonia gas through hydrogen bonding but also assisted in the functionalization of graphene through p-doping (hole rich). This environmentally friendly synthesis strategy can have huge potential of commercialization since it is relatively simple, cost-effective, scalable, and uses biocompatible materials [143].

GO, which is the derivative of PG, is another exciting material for sensor applications since high-quality GO flakes can be produced easily from graphite and can be reduced to prepare highly conductive rGO. Moreover, the zero or quasi zero bandgap of PG can be an impediment for its application as sensitive layer in devices, although this can be overcome by the use of rGO. There are a number of methods used for the preparation of rGO; however, Hummers’ method is by far the most-adopted one and has evolved considerably over the years to produce high-quality (less defective), high-yield, and larger-sized rGO flakes [144,145,146]. In the sub-section below, we will attempt to evaluate the application of rGO in sensors on its own and in conjunction with other nanomaterials, such as conducting polymers, transition metal oxides, and MXene.

**Figure 18:**
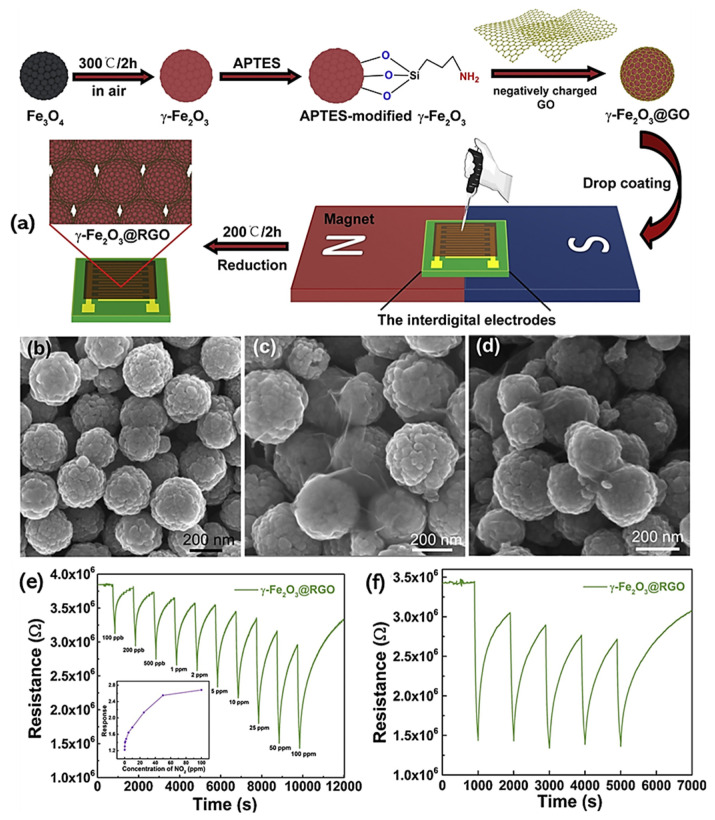
(**a**) Production process of γ-Fe_2_O_3_@RGO-based gas-sensing devices; SEM images of (**b**) γ-Fe_2_O_3_ nanospheres, (**c**) γ-Fe_2_O_3_@GO core-shell hybrids, and (**d**) γ-Fe_2_O_3_@RGO-200 core-shell hybrids; (**e**) variation in sensor resistance under diverse concentrations of NO_2_ in real time; and (**f**) the response replicability under 50 ppm NO_2_. Reproduced with permission from [158].

H_2_ is regarded a valuable renewable source of energy; however, owing to its explosive and flammable nature, it always requires accurate monitoring. Ultrasonic gas sensors commonly based on metal oxides have been employed regularly to monitor H_2_ since these materials have been shown to be highly efficient for H_2_ sensing [147]. However, metal oxides-based sensors require higher operational temperatures between 100 and 200 °C, and their sensitivity can drop drastically with the decline in operating temperatures because of their tiny mass and weak sorption of H_2_. These high-temperature sensors are becoming less desirable for H_2_ sensing applications because of the danger of explosion and higher energy consumption. Therefore, ultrasonic sensors operating at room temperature were given substantial research considerations, with the very first sensor tested at room temperature in a nitrogen atmosphere, since, due to the reaction of oxygen in air with H2, it was extremely challenging to produce and test high-sensitivity sensors in the air [148,149]. In a recent study, Zhang et, al. manufactured an ultrasonic sensor on an rGO-sensitive layer and a 128° YX-LiNbO_3_ substrate using a platinum catalyzer operating at room temperature. The sensor’s sensitivity was increased by adjusting the deposition parameters of rGO, achieving considerably higher sensitivity at room temperature. This improved room-temperature ultrasonic gas sensor was able to detect exceptionally low concentrations of hydrogen of approximately 5 ppm [150]. Similarly, R. Kumar and co-workers also fabricated an rGO-based room temperature gas sensor for SO_2_ detection. The sensor displayed a remarkable sensing response of 3.21% for SO_2_ at a low ppm level of 5 ppm at room temperature, and its sensitivity increased with an increase in SO_2_ level in ppm [151]. GO/rGO is an excellent material for gas-sensing applications; however, there is great room for improvements in the sensing ability of GO/rGO due to its non-dense carbon atom arrangement and lack of selectivity at low/room temperature. Likewise, other nanomaterials such as metal oxide-based nanostructures have immense potential sensing applications; however, these nanomaterials display low sensitivity and operate at considerably higher temperatures resulting in high-power consumption, making metal oxide less desirable in sensing applications. Therefore, the development of a graphene-metal oxide (GO-MO) hybrid and GO-MO interfacial heterojunctions to improve their available specific surface area and enhance their surface adsorption sites, resulting in enhanced sensing performances especially at lower/room temperatures, has been recently studied and is of immense interest [152,153,154,155]. A diverse range of synthesis strategies have been used to prepare rGO-MO composites for sensing applications; however, the drop-coating technique is the most used, although the use of this method can result in nonuniformity due to the coffee-ring effect [156,157]. To avoid the coffee-ring effect, sensing nanomaterial can be deposited on anticipated substrates in a controlled manner using external forces. In a recent study by Zou et al, a 3D γ-Fe_2_O_3_@rGO core-shell film was distributed on the desired substrate by deploying a magnetic field. This core shell film based on 3D γ-Fe_2_O_3_@rGO not only eschewed the undesirable coffee-ring effect but also resulted in heteroatoms (p-type) doping, as well as the introduction of surface defects with enhanced gas-sensing performance. The room temperature N_2_ gas sensor displayed excellent selectivity and superior sensitivity of 3.43 toward 50 ppm of NO_2_, which was two and half times higher than that of the pure rGO sensor. The sensor’s performance stayed remarkably high even at an extremely low level of N_2_ of approximately 100 pbb, with a response value of 1.23 [158]. The sensor’s assembly procedure and its various performance characteristics are shown in Figure 18. 

Figure 18a shows a schematic of the entire production process of the γ-Fe_2_O_3_@rGO-based room temperature N_2_ gas sensor. Figure 18b illustrates the nanocrystal aggregates of Fe_3_O_4_ in the shape of uniform-sized nanospheres, whereas in Figure 18c, it can be observed that there is an insignificant change in the size and uniformity of these spheres after reduction, and lastly, Figure 18d shows the rGO/γ-Fe_2_O_3_ hybrid where it can be witnessed that the Fe_2_O_3_ sphere remained in shape but was covered in a thin layer of rGO sheets with wrinkled features to produce γ-Fe_2_O_3_@rGO core-shell hybrids. Response–recovery curves are recorded in Figure 18e in NO_2_ concentration in the range of 100 ppb to 100 ppm, where fast and excellent sensing can be observed, whereas Figure 18f shows its outstanding stability and reproducibility.

Gas sensors based on conducting polymers (CPs) have also received considerable attention recently because of several advantages such as the ease of synthesis, low/room temperature operability, and stability [159]. Among other CPs, polyaniline (PANI) is frequently adopted for gas-sensing applications particularly due to its sensitivity to a diverse range of gases including NO_2_, NH_3_, H_2_, CO, CH_3_OH, and N_2_H_4_ alongside other superior characteristics of CPs, which are the main reasons for its adoption in gas-sensing applications [160]. However, PANI do have various shortcomings such as relatively low processing ability and mechanical strength. These inadequacies can be overcome, and their gas-sensing properties can be tailored substantially by forming graphene/conducting polymer hybrids [161,162]. Karouei et al. recently studied the gas-sensing characteristics of a graphene/polyaniline nanocomposite under varying humidity conditions at room temperature. Composites prepared with 20 wt% of graphene were found to be the optimum composition for sensitivity, reversibility, and better protonation degree. The gas-sensing performance of CO_2_ of this hybrid was three time better than that of PANI, with excellent long-term stability, where an only 18% drop was observed in its performance after constant use for one year [163]. 

**Figure 19:**
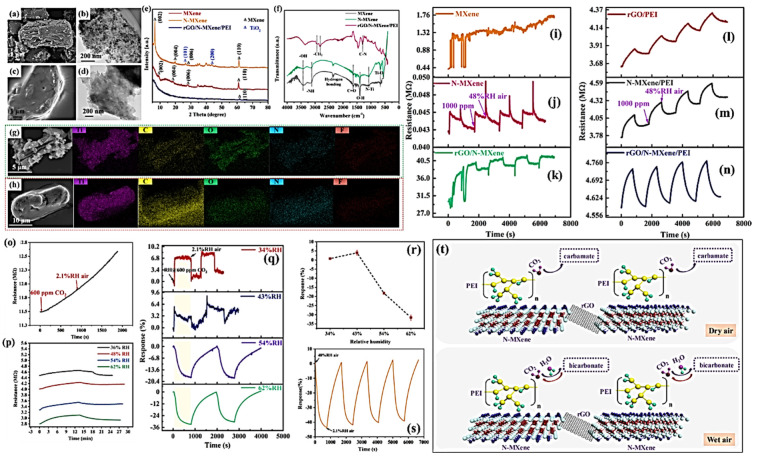
(**a**) SEM image of N-MXene; (**b**) TEM image of N-MXene; (**c**) SEM image of rGO/N-MXene/PEI composites; (**d**) TEM image of rGO/N-MXene/PEI composites; (**e**) XRD spectra; (**f**) FTIR study of MXene, N-MXene, and rGO/N-MXene/PEI hybrid; (**g**) elemental mapping of N-MXene and (**h**) rGO/N-MXene/PEI nanocomposites; resistance transients of sensors (**i**) MXene, (**j**) N-MXene, (**k**) rGO/N-MXene, (**l**) rGO/PEI, (**m**) N-MXene/PEI, and (**n**) rGO/N-MXene/PEI sensors for 1000 CO_2_ in 48% (relative humidity) RH air at room temperature (20 °C); (**o**) dry and (**p**) humidified dynamic resistance of the composite (ternary) sensor at 20 °C under 600 ppm CO_2_; sensing performance of the ternary sensor: (**q**) Response toward a mixture of 600 ppm CO_2_ and under varying RH, response as a function of RH obtained from (**r**–**t**) response to pure 48% RH at 20 °C and schematic representation of the CO_2_ sensing mechanism of rGO/N-MXene/PEI sensors under dry and wet air respectively. Reproduced with permission from [164].

To further enhance the detection limits of gas sensors into the ppm range (trace detection), reduce their operational temperatures (lower power consumption), and improve their suitability for use in exceptional situations (potentially flammable and explosive settings), composites of graphene with other materials such as CPs and TMOs have been used. In some cases, ternary composites are developed, where graphene hybrids are produced with two other sensing materials simultaneously, where the overall performance can be improved significantly when compared with sensors manufactured using individual sensing materials. In a recent study by Zhou et al., rGO, N-doped MXene (Ti3C2Tx), and polyethyleneimine (PEI) ternary hybrids (rGO/N-MXene/PEI) were used as active materials for CO_2_ detections and displayed exceptional sensing performances. By using this hybrid composition, excellent sensing operations were achieved with outstanding detection limits of 8ppm at room temperature (20 °C). This was attained by optimizing various parameters with a PEI concentration of 0.01 mg/mL and an RH of 62%. Experimental results revealed that along with the extraordinary sensing performance of rGO, layered N-MXene offered an abundance of active sites for CO_2_ and water co-adsorption, whereas PEI polymers were suitable for the binding of CO_2_ molecules, resulting in induced appreciable density variation of charge carriers via proton-conduction behaviour [164]. Figure 19 shows various physical characteristics as well as the electrochemical performance of rGO/N-MXene/PEI hybrids as the active material for CO_2_ sensing application. 

Graphene and its derivates such as GO and rGO have already demonstrated excellent gas-sensing characteristics when compared with other pristine structures, e.g., carbon nanotubes (CNTs), metal oxides, and conducting polymers, because of their high surface area, tunable electronic/chemical properties, and chemically active surfaces. However, graphene’s sensing-ability is low for certain gases such as NH_3_ due to weaker bonding and inferior charge transfers between these two materials. Moreover, Graphene has a less dense structure and lacks selectivity. Metal oxide-based sensors can only operate at higher temperatures, making them less safe and more energy intensive. CPs such as PANI have their own shortcomings such as the mechanically feeble structure. Therefore, engineering of nanohybrids through hybridization of graphene and its derivatives with nanomaterials including metal oxides, MXene, and conducting polymers can result in superior sensing properties, and these composites exhibit improved sensing performance compared to their individual constituents due to a synergistic effect, also referred to as non-interface-dependent complementary behavior. Therefore, it is believed that the likely direction for future R&D work on graphene-based gas sensors will focus on combining graphene with other functional nanomaterials with improved activity. In addition, theoretical analyses and computational modellings are also obligatory to identify the underlying principles of different interactions taking place between different gases and graphene-based nanomaterials on their surfaces, thereby shifting the research focus from devices to solid–gas interfaces. 

### 3.2. Membrane Applications

Organic dyes, heavy metal ions, organic solvents, and oil are some of the main pollutants of water resources [165]. Moreover, excessive concentrations of other harmful compounds such as nitrate, fluoride, arsenic, selenium, and sodium can also make groundwater unsuitable for human consumption since they can result in various health implications [166]. Over the years, a number of techniques have been developed to treat and purify waste and groundwater. Some of these techniques include solvent extraction, flotation, precipitation, oxidation, evaporation, adsorption, and membrane filtration [167,168,169]. Because of its cost effectiveness, operational ease, low energy consumption, superior efficiency, and availability of a diverse range of membrane materials, membrane filtration is considered one of the leading techniques used for water treatments. A membrane is essentially a barrier that can be established with the capability to permit desired species to pass through whilst blocking undesired ones. This can be achieved effectively by fine-tuning membrane properties, e.g., optimizing it structure and chemical composition according to the type of species required to be filtered out. Research in the field of membrane technology is progressing actively since it has a number of key real-world applications other than water purification. These applications include desalination, decontamination, and metal removal/recovery. A number of highly porous membranes based on polymeric membranes [170], activated carbon [171,172], organic–inorganic hybrids [173], carbon nanotubes [174], and zeolite [175] have already been developed and are being successfully used. Nevertheless, the requirement to develop membranes with tunability at the atomic scale that can result in restricting the release of containment species according to their molecular sizes is vital. Membranes’ costs and effectiveness are other vital characteristics necessitating the development of new membrane materials [176]. The technological advancements in graphene-based membranes to enhance graphene’s removal capability of pollutant ions can present a breakthrough in its industrial applications. This is possible since structural and surface characteristics of graphene can be fine-tuned according to the desired field of application. The build-up of heavy-metal ions including Zn, Pb, Fe, Mn, Cu, and Cd released from a wide range of industrial processes such as mining, steel plating, battery manufacturing, and fertilizer/pesticide production is considered one of the major sources of the contamination of wastewaters [177]. Unlike organic compounds, these metal ions are not biodegradable and remain active throughout the food chain, posing serious dangers to human health and ecology. Due to their mechanical strength, excellent chemical stability, large surface area, and existence of various hydrophilic surface functional groups, membranes based on graphene are highly desirable for the removal of these metallic ions from water. [178,179]. In a recent study, Hilal and co-workers investigated the used of single to few-layered GO sheets to produce a lamellar GO membrane and succeeded in the extraction and preconcentration of Cd (II), Pb (II), and Cu (II) from industrial wastewater. Under optimized conditions, a detection limit of 1.1 ngL^−1^ was achieved with excellent accuracy between 4 and 5% across five consecutive measurements [180]. 

Commercially used dyes are also very harmful compounds effecting the quality of water resources. Ultrafiltration and nanofiltration membranes with pore sizes smaller than those of dye molecules have drawn enormous interest for dye removal. However, traditional polyamide-based nanofiltration membranes maintain an extremely high level of rejection for divalent salts such as Na_2_SO_4_ and a lower level of rejection for monovalent salts such as NaCl, along with high dye rejections resulting in low salt removal [181]. On the other hand, in addition to their low slat rejections, the dye rejection of ultrafiltration membranes is also too low for an acceptable level of dye recovery. Therefore, the development of membranes with very high dye rejection and negligible rejection of salt is of great interest for the improved removal of dye and high-level recovery of salt, respectively. Reduced graphene oxide can be used as a potential material for dye removal; however, due to lower interlayer distances, its reduced water permeance can result in extremely high salt rejection. To maintain the inter-layer distance of rGO operatable for dye removal, numerous approaches have been used, such as the production of graphene hybrids in conjunction with materials such as metal organic frameworks and carbon nanotube; however, this complicates the production processes even further [182,183]. One way of maintaining these channel sizes is by deliberately keeping rGO in the solvated state, which can result in a low level of salt rejection and very high rejection of dye due to control over channel sizes. In a study of dye desalination of textile wastewater by Huang et al., solvated rGO was prepared where nanochannels of a controlled size ranging between organic dye molecules (Direct Red 80-DR 80) and salt ions (NaCl and Na_2_SO_4_) were produced through premeditated solvation of rGO to attain enhanced desalination. This was achieved through the deposition of an exceptionally thin layer of a solvated rGO microfiltration membrane, and channel sizes were maintained by maintaining a swollen state. This rGO-based membrane resulted in rejecting ∼99% of dye molecules (Direct Red 80-DR 80) whereas almost zero rejection of Na_2_SO_4_ and NaCl was detected, making solvated rGO material suitable for nanofiltration membranes for high levels of salt permeance and exceptionally high levels of dye rejection [184].

**Figure 20:**
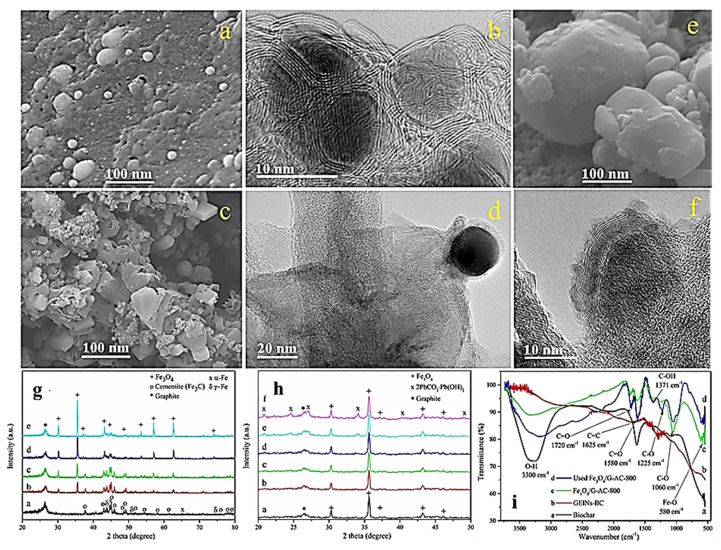
SEM micrographs of GEINs-BC (**a**), HRTEM micrographs of GEINs-BC (**b**), SEM image of Fe_3_O_4_/G-AC-800 (**c**), and HRTEM micrographs of Fe_3_O_4_/G-AC-800; (**d**) SEM (**e**) and HRTEM (**f**) images of Fe_3_O_4_/G-AC-800 after adsorption at pH of 5. XRD patterns (**g**) of graphene-enclosed iron nanoparticles in biochar (GEINs-BC, **a**) and Fe_3_O_4_/G-AC (**b**–**e**) samples activation of GEINs-BC under steam at various temperatures (500, 600, 700, and 800 °C). XRD profile (**h**) of Fe_3_O_4_/G-AC-800 (**a**) before/after adsorption of Pb(II) solution with numerous pH values: pH = 3 (**b**), pH = 4 (**c**), pH = 5 (**d**), pH = 6 (**e**), and pH = 7 (**f**); FTIR profile of biochar (**i**) (**a**), GEINs-BC (**b**), and Fe_3_O_4_/G-AC-800 (**c**) before/after Pb(II) ions adsorption (**d**). Reproduced with permission from [186].

Despite the excellent adsorption performance of carbonaceous martials such as graphene, the removal of a higher quantity of metal ions efficiently from wastewater is challenging. However, the use of composites based on highly porous nanocarbons and magnetic nanoparticles seems to be a viable solution for this purpose, since both materials can complement each other to improve the overall ability of the removal of contaminants [185]. Bhaduri et al. studied this approach, where magnetic/graphene nanocomposites inside activated carbon (magnetic/G-AC) were synthesized and used as adsorbent. The highest adsorption capacity was found to be PH-dependent, and the maximum adsorption was logged at PH5. Figure 20a–f illustrate the SEM and HRTEM micrographs of graphene compressed inside iron nanoparticles in biochar GEINs-BC and Fe_3_O_4_/G-AC-800. It was witnessed that 3–8 nm of crystalline iron nanoparticles were encapsulated inside 2–6 layers of graphene sheets. The XRD pattern in Figure 20g displays a strong diffraction peak at 26.5°, confirming the FLG structure. The different diffraction peaks influenced by cementite Fe_3_C, α-Fe, and γ-Fe are shown in Figure 20g. Figure 20h displays XRD spectra after activation, which show that at an activation temperature of 700 °C, Fe3C, γ-Fe, and α-Fe phases were all oxidized to magnetite. Figure 20i shows the FTIR profile of GEINs-BC, Fe_3_O_4_/G-AC-800, and the used Fe_3_O_4_/G-AC-800 samples. There are no apparent peaks showing heteroatoms on the surface, since heteroatoms could be removed due to the catalytic graphitization process beyond 800 °C [186]. 

**Figure 21:**
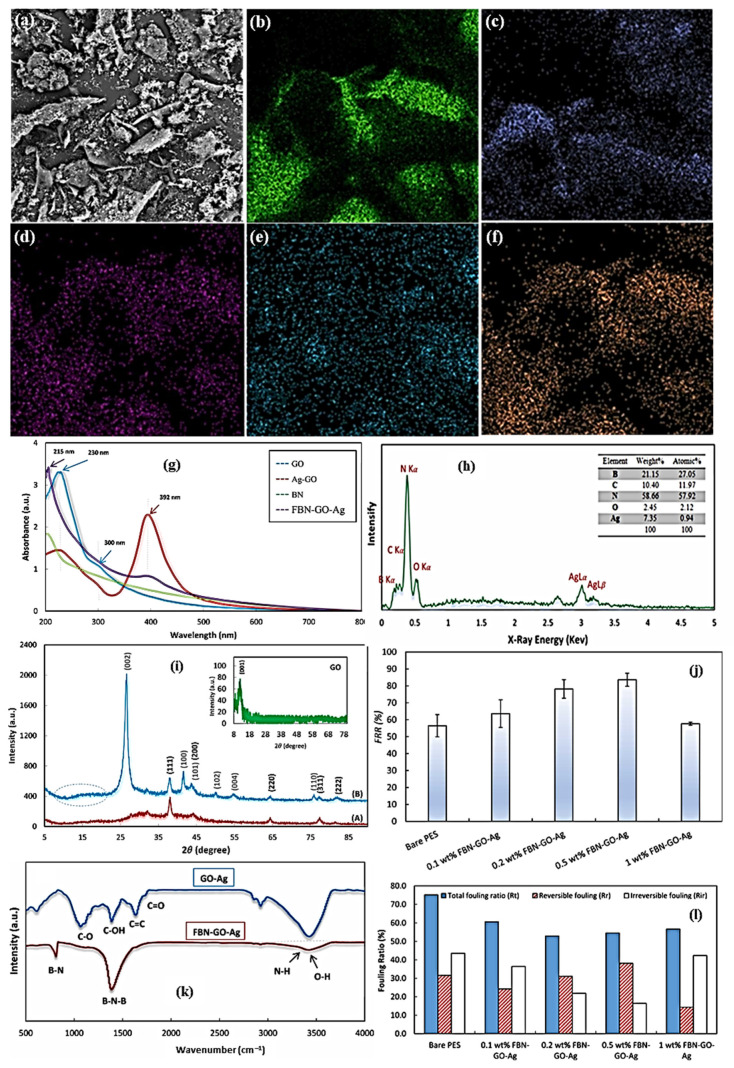
(**a**) SEM micrograph of BN/Ag/GO nanocomposite. (**b**–**f**) Elemental mapping of BN/Ag/GO nanohybrids for different elements of (**b**) C, (**c**) N, (**d**) B, (**e**) Ag, and (**f**) O; (**g**) UV–vis absorption pattern of GO, Ag-GO, BN, and BN/GO/Ag dispersions; (**h**) EDX pattern of the synthesized FBN-GO-Ag nanocomposite; (**i**) XRD profile of (A) GO/Ag nanohybrid and (B) BN/GO/Ag nanocomposite and XRD spectra of GO (inset). (**j**) The flux recovery ratio of FBN-GO-Ag blended PES membranes; (**k**) FTIR spectra of the synthe-sized FBN-GO-Ag and GO-Ag; (**l**) The fouling ratios of FBN-GO-Ag blended PES membranes. Reproduced with the permission from [192].

As discussed above, despite the number of advantageous characteristics of graphene and its derivatives, graphene still struggles to perform efficiently as a membrane as a single material, therefore composites of graphene with other functional nanomaterials such as polymers are an interesting concept. Aromatic polyimides are preferred materials to make hybrids structures with graphene as compared to aliphatic and semi-aromatic polyimides due their nontoxic nature coupled with excellent strength and stability. In a recent study by Zhang et al., a reduced graphene oxide (rGO) and aromatic polyimide (PI) composite (rGO-PI) was synthesized and used as a separation membrane. The substrate of the nanofibrous PI membrane was prepared using a low-temperature polycondensation reaction followed by electrospinning, whereas rGO was produced using a modified Hammer’s method and the entire procedure was performed in a fluorine-free environment. This hybrid membrane attained exceptionally high separation efficiency of over 99% and flux of up to 2040 Lm^−2^h^−1^, which resulted in the effective purification of contaminated water with oil. Moreover, this composite membrane demonstrated tremendous stability under a harsh environment, both chemically and physically [187]. Advancement in the separation field coupled with ground-breaking developments in 2D nanomaterials has revolutionized this area of research, which can help in elevating he freshwater crisis that seems imminent for future generations by using seawater as a water source. Three-dimensional stocking of graphene can also create nanochannels for the efficient transportation of mass, where the functionalization of graphene oxide with heteroatoms of nitrogen and oxygen can not only provide stable dispersibility and surface negativity, but also can assist in further surface modifications. However, the implementation of graphene oxide as a membrane for the purification of seawater is still a challenge because of the reduced rejection of smaller ions, which is caused by swelling (increased d-spacing) of the graphene membrane inside aqueous solutions. This deficiency can be addressed and overcome using various surface modifications. In a study by Qian et al., an N-doped graphene oxide membrane was produced by the one-step plasma process. Both polarized nitrogen atoms and amine groups were introduced on the surface of the membrane and were controlled by plasma processing time. This functionalized graphene oxide-based membrane exhibited an ideal mono/divalent cation selectivity of up to 90 and retained an adequate binary cation selectivity of up to ∼28, which was credited to the robust electrostatic interactions between metal ions and nitrogen functional groups. The graphene oxide-based membrane has also shown a low permeability to salt solutions of less than 0.03 mol m^−2^h^−1^ with a higher water flux of up to 120 mol m^−2^h^−1^, showing very high water/salt selectivity at 4.31 × 10^3^ [188]. Polymeric membranes are normally used for wastewater treatment due to their simplicity, cost-effectiveness, efficiency, and low energy usage [189]. However, extension in d-spacing inside organic solvents, drastic aging in a harsh chemical atmosphere, and the fouling phenomenon are some of the major drawbacks of these types of membranes, with fouling being another main limitation since it can result in inferior membrane performance, flux alleviation, and shortening of the overall lifetime of the membrane [190]. A number of techniques, including polymer blending, coating, and grafting, have been used to alleviate the fouling phenomenon in membranes. However, to address this matter in a facile manner, mixed-matrix membranes (MMMs) are produced since the hydrophobic nature of polymers is considered to be the key cause of fouling, which can be addressed in MMMs through surface modification with the introduction of hydrophilic elements on the membrane surface. The incorporation of 2D materials into MMMs is considered a useful strategy since these can act as a surface shield and can improve membrane selectivity. In addition to surface fouling, biofouling is another issue with these membranes, which can be resolved using materials such as Ag due to its antibacterial properties; however, Ag can result in agglomeration and can cause toxicity. GO can be a key supporting 2D material, which not only alleviates agglomeration through functionalization but also reduces the toxicity of Ag. Furthermore, its negative surface charge improves the antibacterial characteristics of these polymeric membranes, reducing biofouling [191]. A polyethersulfone (PES)-based membrane was fabricated during a research study by blending nanohybrids of 2D functionalized boron nitride/graphene/oxide/silver (BN-GO-Ag) to enhance the separation performance through reduced fouling, increased antibacterial activity, and enhanced permeability of this polymeric membrane. The addition of 1 wt% of BN-GO-Ag resulted in increased hydrophilicity with a decrease in the H_2_O contact angle of the membrane from 61.9° to 48.8°. Decreases in the reversible fouling and fouling rates of 27.2% and 22.2%, respectively, were observed after the addition of 0.5 wt% BN-GO-Ag to the membrane in reversible fouling resistance and the total fouling rate of the synthesized membrane, respectively. Additionally, the PES membrane with 1 wt% BN-GO-Ag nanocomposites exhibited 77.7% and 88.9% rejection of reactive red 120 and reactive black 5, respectively, which can be credited to the increase in surface negativity of the membrane due to the addition of GO [192]. Figure 21 signifies the physical/chemical properties and membrane performance of ternary composites. Figure 21a shows SEM micrographs of the BN/Ag/GO hybrid displaying nano-flakes of GO, whereas BN/Ag particles are also present on the surface of GO, where smaller particle sizes of both BN and Ag can be seen on the surface, which may contribute to the higher level of porosity, providing higher permeability and a large number of active sites. Figure 21b–f represent the elemental mapping of BN/Ag/GO nanohybrids for different elements, i.e., (b) C, (c) N, (d) B, (e) Ag, and (f) O. Figure 21g displays the adsorption peaks for substances of BN, GO, GO/Ag, and FBN/GO/Ag when UV-Vis analysis was performed, where the symmetrical shape of the UV-Vis absorption peak implies a smaller size of the silver nanoparticles and a uniform distribution. Furthermore, Figure 21i presents XRD spectra of (A) the GO-Ag nanohybrid and (B) FBN-GO-Ag samples and the XRD pattern of GO (inset), confirming the surface functionalization of composites and anchoring of Ag and BN particle on GO surface. Figure 21k shows the FTIR profile confirming the existence of oxygen heteroatoms on the surface of ternary composites, which are also confirmed by EDX analysis in Figure 21h. Furthermore, Figure 21j presents the antifouling performance of the membrane, which displays enhanced flux recovery and better anti-fouling properties when compared with the bare membrane, and finally, Figure 21l shows that the resistance factor in altered membranes is lower, even though their flux recovery rate is much higher [192]. 

In summary, graphene with a unique single-atom-thick structure and excellent mechanical and chemical stability has shown some extraordinary permeation characteristics. Graphene membranes can be produced as composites with other materials such as polymers, MXene, or inorganic compounds, and as membranes containing oxygen and nitrogen heteroatoms. However, the synthesis of facile and large-area graphene with controlled layer thickness remains a huge challenge. Another constraint for the use of graphene in membrane applications is the production cost involved to synthesize good-quality graphene. Scalability is another issue that requires attention since larger quantities of active materials are required for membrane applications and is still considered a key hurdle in using graphene for membrane separation applications. Furthermore, graphene production processes are still very complicated. Therefore, further research and development is essential for state-of-the-art membrane production process developments to cost effectively manufacture graphene at a larger scale using simplistic methods. Finally, further investigative work is required to understand the mass transport phenomenon and impact of various parameters such as interlayer spacing, functionalization, and selective defects. This can only be achieved using advanced characterization techniques together with simulation tools. 

### 3.3. Metal Ions Detection Applications 

**Figure 22:**
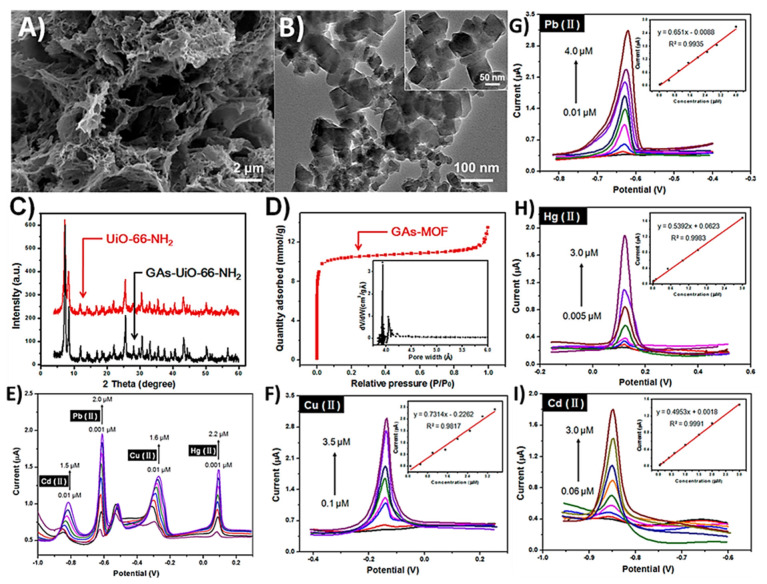
SEM image of (**A**) GAs, TEM image of (**B**) UiO-66-NH_2_, (**C**) XRD spectra, (**D**) N_2_ adsorption-desorption isotherm of the GAs-UiO-66-NH_2_, (**E**) DPSV response of the GAs-UiO-66-NH_2_ altered GCE after adding diverse concentrations of Cd^2+^, Pb^2+^, Cu^2+^, and Hg^2+^ in acetate buffer solution (0.2 M, pH 5.0, DPSV response of the GA-UiO-66-NH_2_ altered GCE for specific analysis of (**I**) Cd^2+^, (**F**) Cu^2+^, (**G**) Pb^2+^, and (**H**) Hg^2+^ over a concentration range of 0.06−3.0 μM, 0.01−4.0 μM, 0.1−3.5 μM, and 0.005−3.0 μM, respectively. Reproduced with the permission from [198].

Metals ions including Hg^2+^, Cu^2+^, Pb^2+^, Ag^+^, and Cd^2+^ are released during manufacturing processes and agricultural activities, which can be highly contaminating and are found in abundance in water resources and wastewaters [193,194]. These heavy metals require precise detection and removal since they not only effect environment and biological ecosystem, but also human health, and these metal ions are nondegradable, unlike organic compounds. Therefore, it is imperative to develop highly efficient analytical techniques to detect and quantify heavy-metal concentration in samples of different matrices. There are a number of very sensitive systems have been developed and deployed for heavy metal ion detection such as X-ray fluorescence spectrometry (XRF), atomic absorption spectrometry (AAS), atomic emission spectrometry (AES), and inductively coupled plasma mass spectrometry (ICP-MS) [195]. Nevertheless, these methods are labor intensive, time consuming, and costly, necessitating effective alternatives. Sensing technology is growing at a steady pace where graphene, graphene derivates, heteroatom-doped graphene, and composite-based sensors are being widely developed [196,197]. A range of graphene-based sensors have been developed over time in different areas; however, optical and electrochemical sensors are extremely important for environmental protection applications in general and heavy metal ion detection in particular. Electrochemical sensors are of particular interest since these are easier to use, cost-effective, have extremely low detection limits, and, most importantly, these can be used to detect multiple heavy metal ions simultaneously. Graphene has been widely utilized as the active material for electrochemical sensors on its own or in conjunction with other materials including metal oxides, activated carbons, and metal organic frameworks (MOFs). In a recent study by Lu et al., graphene aerogels (GA) and a UiO-66-NH_2_ MOF composite was utilized as an electrode material for the detection of multiple heavy metal ions in a real-time study. The solvothermal method was adopted for the in-situ growth of UiO-66-NH_2_ on graphene surface. The key motive for the synthesis of this composite was to enhance the conductivity of the MOF-based composite since MOF has excellent properties such as structural tuneability, high surface area but are electrically nonconductive materials. TEM, SEM, and XRD were employed to characterize the structure and morphology of GA/MOF composites whereas the porous structure was analyzed using nitrogen adsorption/desorption analysis. Figure 22A shows that GA retained an interconnected network structure with opened pores, which can provide a large active surface area for functional material, whereas Figure 22B shows TEM images displaying random accumulation of UiO-66-NH_2_ with octahedral cubic intergrown morphology. Figure 22C displays the XRD profile of UiO-66-NH_2_ and GA-UiO-66-NH_2_ composites exhibiting an analogous XRD pattern for both samples confirming the successful integration of UiO-66-NH_2_ into graphene, where the GA template and matrix, as well as the integrity, of UiO-66-NH_2_ were not damaged by GA. Figure 22D presents the nitrogen adsorption/desorption isotherm where large nitrogen intake at lower relative pressure is indicative of microporosity, which is also confirmed by the pore size distribution (inset). The calculated specific surface area was 707.79 m^2^g^−1^, which will provide a large number of active sites and mass transport channels. As shown in Figure 22E, the stripping peak currents of different metal ions increased with the rising metal-ion concentrations. Figure 22E–I show the DPSV response of the GA-UiO-66-NH_2_-modified GCE for individual analysis, with Cu^2+^, Pb^2+^, Hg^2+^, and Cd^2+^ over a concentration range of 0.06−3.0 μM, 0.01−4.0 μM, 0.1−3.5 μM, and 0.005−3.0 μM, respectively. These results revealed that the response current of every metal ion was linearly enhanced when boosting target analyte concentrations [198].

Even though graphene has prevailed as a wonder material and a potent platform for diversified fields including electrochemical sensing applications, 𝜋–𝜋 stacking and weak interactions can result in restocking/agglomeration of graphene layers resulting in reducing interlayer spacing. This can be addressed through surface functionalization or by producing composites using other nanomaterials such as conducting polymers and metal oxide nanoparticles [199,200]. In a recent work, Akhtaret al. designed an effective electrochemical sensor comprising rGO covered with alanine and polyaniline (rGO/Ala/PANI/GCE) by compounding the electro-catalytic and chelating properties of alanine and polyaniline alongside graphene for its increased surface sites, high charge exchange kinetics, and superior conduction properties. Under optimized conditions, the functionalized sensor demonstrated excellent stability, sensitivity, and selectivity. Physiochemical characterization was performed using state-of-the-art techniques including XRD, UV-vis, FTIR, and SEM confirming successful production, whereas electrochemical characterization was performed using CV and EIS measurements. The produced sensor based on rGO/Ala/PANI/GCE composites displayed an extensive linearity range of 100 nM–0.08 nM and extremely low detection limits of 0.03 nM, 0.045 nM, and 0.063 nM for Cd^2+^, Pb^2+^, and Cu^2+^ ions, respectively. These are attributed to the exceptionally high surface binding attraction, enhanced electrical conduction path, electron tunnelling, and ion-trapping characteristics along with the extraordinary electrocatalytic activity of rGO/Ala/PANI composites [201]. As discussed above, due to its superior properties, graphene can be used for electrochemical sensor applications; however, using graphene on its own is still extremely challenging due to the restocking and agglomeration of graphene layers. Therefore, the use of functionalized graphene oxide/reduced graphene oxide or composites in conjunction with other nanomaterials are preferred, as they can serve as a composite precursor and support material. 

Other most commonly used sensors for heavy metal ions detection include optical sensors. In optical sensors, a change in light beam is measured, which occurs due to the alteration in the intensity of light. This is due to variation in the light optical characteristics, e.g., wavelength, phase, spectral distribution, and polarization. Optical sensors are one of the most versatile tools to detect changes in a wide range of characteristics including pressure, temperature, radiation level, and chemical concentrations. In the past, low-dimensional (zero- and one-dimensional) materials such as quantum dots, carbon nanowires, gold nanoparticles, and nanotubes had been used intensively in optical sensor applications [202]. However, the discovery and development of graphene and its derivates/composites have resulted in its increased use in optical sensing applications because of graphene’s outstanding characteristics, for instance, its excellent biocompatibility, robust chemical stability, large specific surface area, mechanical strength, ease of production, reduced cost, and ability to absorb biomolecules through π-π stacking [203,204].

Cobalt (Co^2+^) is a heavy metal present in the human body in the range of 1–2 mg, which is an essential part of vitamin B_12_ that is necessary for the functioning of various body organs including the liver, kidney, heart, and brain. However, an excess of Cobalt (Co^2+)^ in the human body can result in various health complications including higher heart rates, asthma, headaches, and fibrosis in the lungs, consequently requiring accurate monitoring [205,206]. In a recent study by Daniyal et al., a surface plasmon resonance (SPR) optical sensor was prepared using chitosan–graphene oxide-based composite thin films for its prospective application in the detection of cobalt ions (Co^2+^). Thin films were characterized by XPS to confirm the chemical interactions involving Co^2+^ with existing functional groups on the surface of thin film. These thin-film optical sensors could detect very low concentrations of Co^2+^ of as low as 0.01 ppm [207].

Graphene-based nanomaterials and their composites have been extensively investigated for heavy metal ion detection with high sensitivity and selectivity. However, PG is not a preferred choice for sensing applications due to the issue of π-π stacking and layer accumulation, therefore functionalized graphene or binary/trinary composites with other nanomaterials such as metal oxides, conducting polymers, and MOFs are the preferred choice for sensing applications. Since research in the field of metal sensing using graphene and its derivates/hybrids is still in very early stages, further investigative work is required to improve the detectability of graphene and its hybrids for wider deployment of graphene in these sensing applications. 

### 3.4. CO_2_ Conversion Applications 

One of the main reasons of global warming is the amount of carbon dioxide (CO_2_) in the atmosphere and its exponential growth at a disturbing rate, which results in the greenhouse effect [208,209]. The wild emission of industrial gases, tremendous use of fossil fuels, and deforestation have prompted rising atmospheric CO_2_ concentrations considerably in the past few decades. The extent of CO_2_ utilization by organic chemicals is somewhat smaller than the CO_2_ produced through the burning of fossil fuel [208,210]. The atmospheric CO_2_ grasps a large amount of solar heat energy, enacting feasible obstacles for excess heat to leave the upper berth atmosphere, leading to a rise in the global temperature and thereby greenhouse gases in the air, which impact natural life on earth [211,212]. To defeat the unfavorable concerns of global warming, significant research has been dedicated to the development of materials that can adsorb CO_2_. The precise and reliable conversion of CO_2_ into useful chemicals also helps to minimize CO_2_ emission and waste gas utilization [213]. Nowadays, various CO_2_ capture and storage technologies are available commercially, which not only reduce atmospheric CO_2_ levels, but also store it and allow for their conversion into very useful chemicals as well as organic materials thereafter [214,215]. The research on CO_2_ conversion into different chemicals and organic materials is also crucial for sustainable developments, and therefore CO_2_ consumption and conversion constitute one of the biggest steps of CO_2_ recycling. Different strategies have been employed to convert CO_2_ into organic materials, such as photocatalysis, electrocatalysis, photo-electrocatalysis, etc. [216]. Catalytic conversion of CO_2_ has now attracted significant attention from scientist and researchers since it can contribute to the carbon cycle balance and produce different valuable chemicals [217,218]. CO_2_ can be transformed into different useful gases and fuels, such as CO, CH_4_, CH_3_OH, and HCOOH, as well as various hydrocarbon-based fuels [219,220]. In addition to this, catalytic conversion of CO_2_ into useful chemicals such as carbonates, carboxylates, carbamates, etc,. is also desirable [221]. The synthesis of organic cyclic carbonates, electrolytes for rechargeable batteries, and polar aprotic solvents constitutes one of the large-scale industrial transformations of CO_2_.

Usually, the conversion of CO_2_ reactions involves high bond energy because of its chemical inertness and high C=O bond energy of the CO_2_ molecule. The traditional CO_2_ conversion processes are performed at very high temperatures and under high-pressure conditions, which utilize more energy that the energy of produced fossil fuels as heat resources. Different homogeneous catalysts such as noble-metal catalysts (Au, Pd, Cu, and Ag,) have been employed for CO_2_ conversion; nevertheless, the preparation of homogeneous catalysts has always been a challenge. Furthermore, there are some issues associated with their higher costs, high overpotential, and sensitivity, and these restrict their large-scale commercialization. Therefore, in recent years, heterogeneous catalysts such as carbon-based materials (graphene, CNTs, carbon spheres, and activated carbons) have been considered promising candidates with excellent activity and low costs for CO_2_ conversion applications [222]. Graphene and graphene-based materials with outstanding chemical and physical properties and high specific surface area have attracted significant attention and research interest as advanced catalysts for CO_2_ conversion. The structure of graphene consists of different oxygen heteroatoms such as C=O, C=OH, and COOH, which can function as highly active sites for different catalytic reactions such as reduction, oxidation, hydrogen evolution, and coupling. Different functional groups of graphene oxides are typically acid or base sites, which function as promising heterogeneous catalysts for different conversion reactions.

**Figure 23:**
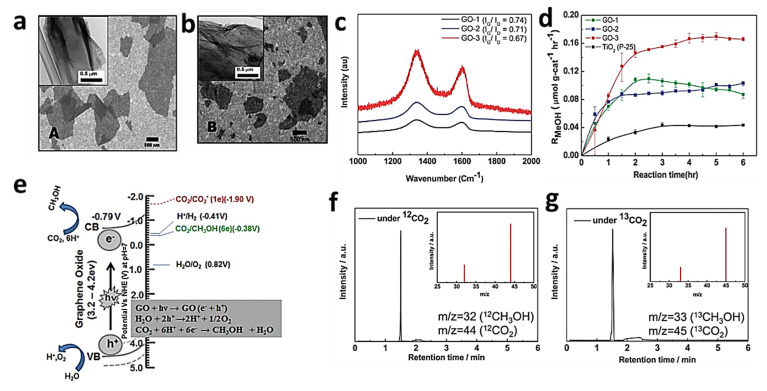
(**a**,**b**) SEM images of GO sheets on gold substrate. (**c**) Raman spectra for three different GO sheets, (**d**) photocatalytic methanol formation (RMeOH) on various GO sheets and TiO_2_ using simulated source of solar-light, (**e**) schematics showing the photocatalytic CO_2_ reduction mechanism on GO sheets, (**f**,**g**) MS chromatograms and profiles of methanol produced by photocatalytic reduction of ^13^CO_2_ or ^12^CO_2_ with 0.2 g GO-2 with (**f**) MS chromatogram at *m*/*z* 32 in ^12^CO_2_ and (**g**) MS chromatogram at *m*/*z* 33 in^13^CO_2_. Reproduced with the permission from [223].

Hsu et al. [223] performed the photocatalytic conversion of CO_2_ into methanol, employing different GOs as the catalysts. Three different GOs were prepared using a revised Hummer’s method by adjusting the oxidant KMnO_4_ and H_3_PO_4_. The SEM micrographs of GO sheets are presented in Figure 23a,b, illustrating few layered flakes. The Raman spectra in Figure 23c for all GO samples shows a strong G mode (1590 cm^−1^) and D mode (1590 cm^−1^), respectively. Photocatalytic CO_2_ reduction was performed at room temperature using a gas flow reactor and a halogen lamp light source, where CO_2_ was expelled inside, and the methanol concentration was determined through GC-FID in the vapor phase. The methanol formation with the reaction time is plotted in Figure 23d. The CO_2_ to methanol conversion rate with GO-3 as the catalyst was 0.172 mmol g cat^−1^ h^−1^. The photocatalytic CO_2_ reduction using GO is schematically illustrated in Figure 23e. During the photocatalytic CO_2_ reduction, photogenerated electrons (e−) and holes (h+) travel to the GO surface and function as reducing and oxidizing sites. The reduction potential of e− (donor) in the GO conduction band was −0.79 V (vs. NHE) (smaller than CO_2_/CH_3_OH, −0.38 V vs. NHE) and the oxidation potential of h+ (acceptor) in the GO valance band was nearly 4 V (higher than H_2_O/O_2_, 0.82 V vs. NHE). The photogenerated e^−^ and h^+^ on the GO surface react with adsorbed CO_2_ and H_2_O and produce methanol via the reaction shown in Figure 23e. A mass spectroscopy analysis further confirmed the methanol formation via CO_2_ reduction as shown in Figure 23f,g. As is known, GO contains very thin layers of interconnected carbon atoms. The hybridization angles of carbon atoms in GO are 90° and 120°, which cover various oxygen functional groups [224]. The reduction of GO to rGO alters the oxygen content in oxygen functional groups, leading to an improved electrochemical performance in CO_2_ reduction. Gusain et al. [225] successfully developed a reduced graphene oxide-copper oxide (rGO-CuO) composite for the photocatalytic conversion of CO_2_ into methanol. The bare CuO nanomaterials showed low photocatalytic activity as they absorb visible light well and create electron–hole pairs. The electron–hole pairs recombine quickly prior to the photocatalytic reaction because of the lower band gap of CuO, which yielded only 175 μmol g^−1^ of methanol. Besides, rGO–Cu_2_O and rGO–CuO composites illustrated enhanced photocatalytic activities as the photogenerated electrons in the conduction band of CuO were effortlessly transported to the rGO network, leading to the slow recombination of charge carries and effective transportation of photo-generated electrons to the catalytic sites of rGO network, which reduce adsorbed CO_2_ into methanol. The rGO–CuO and rGO–Cu_2_O composites showed 1228 μmol g^−1^ and 862 μmol g^−1^ of methanol, respectively. Similarly, An et al. [226] proved that the rGO coating on Cu_2_O enhances the photocatalytic activity of the Cu_2_O/rGO composite six times more compared to pristine Cu_2_O.

**Figure 24:**
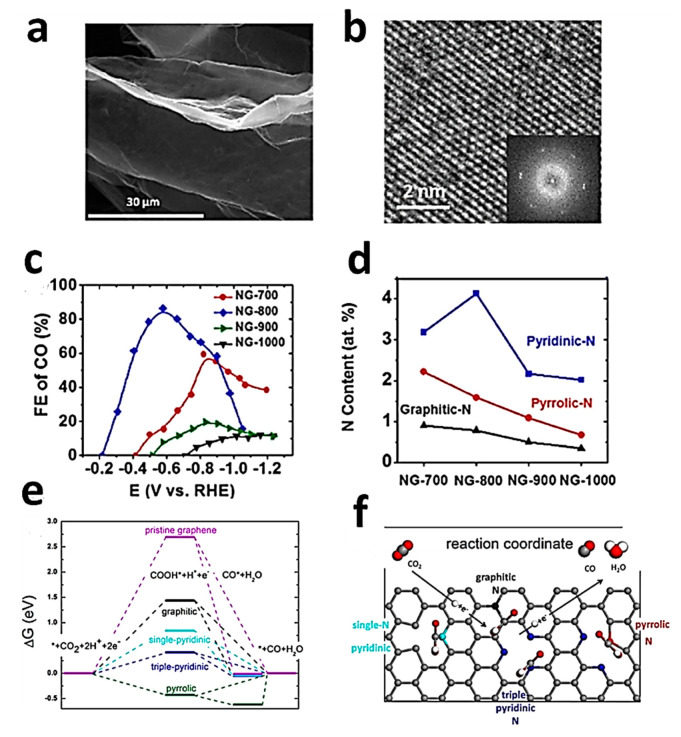
(**a**,**b**) SEM and TEM images of N-doped 3D graphene foam. (**c**) Faradaic efficiency of CO production vs. potential on N-doped 3D graphene doping temperatures of 700–1000 °C. (**d**) The corresponding N functionality content. (**e**) Free energy diagram of electrocatalytic CO_2_ reduction on N-doped 3D graphene and (**f**) schematic of N configuration and CO_2_ reduction mechanism. Reproduced with the permission from [234].

Furthermore, the electrochemical conversion of CO_2_ into different commercially valuable products has been reviewed and has shown to be advantageous owing to its simple operation at ambient temperatures [227]. Different products such as methane, ethane, ethylene, methanol, ethanol, formic acid, and amines have been prepared through the employment of different electrochemical reactions under certain parameters [208,228]. Zarandi et al. [229] developed an electrocatalyst using platinum nanoparticles on histamine-rGO (Pt@His-rGO) to reduce CO_2_ into methanol using electrochemical reduction. Histamine is an electron-rich heterocyclic compound that functionalizes rGO and improved its electrocatalytic activity for the CO_2_ adsorption. Additionally, the Pt nanoparticles on rGO can offer the essential hydrogen radicals for the reduction process. The 0.1 mol L^−1^ KNO_3_ saturated solution with CO_2_ at pH 2.0 electrolyzed on Pt@His-rGO/GCE at −0.30 (vs. Ag/AgCl) for 16 h produced 2.96 mmol L^−1^ methanol. The Faradaic efficiency of 37% was obtained for methanol production. Zhang et al. [230] also used GOs as heterogeneous catalysts for the conversion of CO_2_ into cyclic carbonate. GOs allowed 97.8% styrene oxide conversion with 97.4% chemo-selectivity to phenylethylene carbonate over the 12 h reaction time. From kinetic and XPS studies, it was proven that GO catalysts with a large number of oxygen heteroatoms deliver higher reaction rates. The performance efficiency was greatly altered with surface characteristics of different GO catalysts for the cycloaddition reaction. N-doped graphene had been popular for the electrochemical reduction of CO_2_ into different useful chemicals including CO [231], HCOO^−^ [232], and CH_4_ [233]. The pyridinic-N and pyrrolic-N species from N have stronger CO_2_ adsorption ability. Wu et al. [234] incorporated N-defects in 3D graphene foam and used it as a catalyst for CO_2_ reduction. The SEM images shown in Figure 24a revealed 3D microporous structure of N-doped 3D graphene, and the TEM images in Figure 24b proved few-atomic-layer structures. The integration of N into the graphene compound lowers the energy barriers for the formation of *COOH and enables CO production, as shown in Figure 24c,d. The electrochemical reduction of CO_2_ to CO generation on different N-doped graphene and bare graphene free energy diagrams via the lowest-energy-consuming route are presented in Figure 24e. Additional overpotential is introduced as the uphill barrier of the first electron-transfer rate-determined step for *COOH production. *COOH has great affinity with N defects. The free energy barrier for *COOH adsorption is considerably reduced on pyridinic- or pyrrolic-N species of N, as shown in Figure 24f. The N-doped 3D graphene foam showed very small overpotential of −0.19 V for CO_2_ reduction and formation of CO, which is outstanding compared to noble metal catalysts such as Au and Ag. The N-doped 3D graphene foam delivered 85% faradaic efficiency at an overpotential value of −0.47 V for CO formation and excellent stability for 5 h.

Although graphene has seen increased used in CO_2_ conversion and storge applications, it still requires an immense amount or R&D activity to bring it in line with other heavily used techniques. Even though those techniques are costly, they are proven to work.

### 3.5. Trend Analysis Graphene Use in Environmental Applications 

Trend analysis was also performed by extracting and analyzing data on the above-discussed applications and comparing them with overall environmental applications of graphene. An upward trend in publications in all four areas of environmental applications of graphene was observed, which validates the growth in the use of graphene in these applications and also in overall environmental applications over the past decade. 

**Figure 25:**
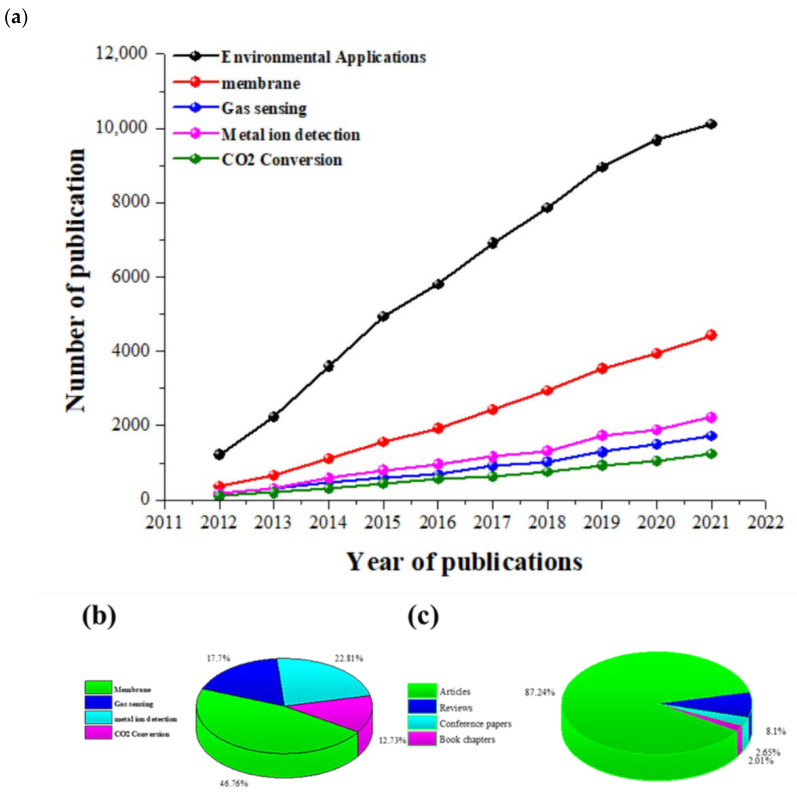
(**a**) Publication trend of graphene in environmental applications from 2010 until 2021, (**b**) The percentage publications graphene in each environmental application, (**c**) Type of publications of the environmental application of graphene. All self-extracted from Scopus (www.scopus.com) (accessed on 11 September 2022).

As can been seen from Figure 25a,b, the use of graphene in environmental applications is growing steeply, with its utilization in membrane applications leading the way followed by metal ion detection. Graphene and graphene-based materials possess outstanding physiochemical properties that lead to a significant research interest in graphene as an advanced material for membrane applications, since for membrane applications, properties such as the tunable structure, modification of surface chemistry through functionalization, and synthesis of a wide variety of hybrid structures are some of the fundamental motivations behind higher research activity in this area while using graphene as an active material. Whereas, in gas sensing and CO_2_ conversion applications, other nanomaterials are leading and currently being used successfully, which shows the requirements for an immense effort to commercialize graphene for these applications by shifting the results of graphene research on these applications outside laboratory settings and replacing the traditionally used materials with graphene. As with graphene synthesis, a trend analysis of graphene applications, shown in Figure 25c, indicated that most of the published data are on experimental work, giving the indication that immense research is underway to investigate and utilize graphene and graphene’s derivative/hybrids in these applications. 

Although immense progress has been made for the use of graphene in environmental applications, it will still be an enormous challenge for years to come to make lab-based research suitable for wider commercial applications. 

As discussed above, graphene, its derivates, and composites show an adequate capability for different applications such as environmental applications, sensing applications, membrane materials, metal ion detection, and gas sensing/storage. However, there are a number of other environmental applications such as bio/chemo sensors, flexible electronics, bio-culturing, electrocatalysis, and photocatalytic degradation of pollutants in which graphene has shown apt capabilities and performances; however, these cannot be discussed in detail in this review article due to its limited scope and size constraints [46]. Photocatalytic degradation of pollutants using graphene has seen immense research interests recently, where pollutants are oxidized using advanced oxidation processes (AOPs) making these contaminants less harmful or even harmless, since photocatalysis can be performed under ambient conditions using sunlight as a natural source of energy. Therefore, this technique has also recently been witnessing huge attention, even though research in this field dates back to the 1960s [235]. This process uses renewable energy in the form of sunlight, with less harmful or harmless by-products such as CO_2_ and water produced. Furthermore, graphene has been widely adopted in photocatalysis degradation applications due to its large specific surface area and tunable porous structure, which can provide a large number of active sites for the photocatalytic process to occur. Graphene has mostly been used in the form of composites, providing support to other materials, and acting as a host material to active materials such as metal oxides and semiconductors in the photocatalytic degradation of pollutants [236]. Since its successful isolation from graphite layers in the early 2000s, a number of new applications for graphene are constantly emerging, and this trend will continue for years to come. The trajectory of the research on graphene and its derivatives/hybrids shows that this material, with its exceptional physiochemical properties, will see many applications in a diverse range of research areas, particularly in environmental applications.

## 4. Conclusions and Outlook

Over the past two decades, efforts have been made to produce high-quality graphene, its derivatives, and composites with an intent to reduce costs and make its production processes simpler. It is anticipated that this trend will continue at a fast pace to develop new synthesis procedures and modify the existing ones, unfolding new and interesting properties of graphene and graphene-based nanomaterials while broadening their applications. A number of production processes have been developed and used, however these synthesis approaches have their own benefits and shortcomings. For instance, chemical vapor deposition has immense potential for commercial-scale applications and is currently the most viable and widely used technique since it results in producing large-sized and high-quality graphene with a controlled number of layers. However, this method still requires the development of an efficient transfer process. Exfoliation methods, whether liquid, chemical, or mechanical, are much simpler, low-cost, and scalable processes to synthesize graphene, not requiring post-production transfer. However, controlling the size and number of layers of graphene flakes is extremely challenging while using exfoliation-based synthesis strategies, and as shown by trend analysis data, exfoliation-based synthesis techniques have seen increased scientific consideration over the past few years. Since the discovery of graphene, the community of material scientists has been facing an immense challenge to cost-effectively produce superior-quality graphene with a controlled number of defects and layers using environmentally friendly processes, since the performance of graphene-based devices greatly depends on these production parameters. It is predicted that applications such as sensing, in which a small quantity of active materials is needed but high-quality graphene is required, will be fulfilled via the chemical vapor deposition method, whereas applications where a higher quantity of material is necessary will be addressed by one of the exfoliation methods. However, with rapid developments in exfoliation-based techniques, it is likely that scientists would be able to modify this synthesis technique to produce substantial quantities of good-quality graphene tailored to a wide range of applications.

Graphene has been used in a large number of environmental applications, whereas in this review, we have focused on four of its key applications, i.e., membrane, gas sensing, heavy metal ions detection, and CO_2_ conversion. Each of these applications required finetuning of its different parameters. For example, graphene’s application as an efficient adsorbent requires a high level of porosity, control over the porous structure, and hydrophilic interactions with pollutant ions. Pristine graphene struggles to fulfil all these requirements, whereas its derivatives and composites not only improve its surface area but also enhance its surface chemistry for improved adsorption of pollutant ions. Therefore, the production and use of graphene’s derivatives and hybrids is highly desirable when graphene is used as an adsorbent. Similarly, graphene has seen increased interest in its application in gas sensing, since, by using graphene for gas-sensing applications, a low operational temperature and improved surface activities can be attained. These are some of the fundamental challenges faced by sensing technology used commercially at present. These requirements can be easily achieved with the use of graphene, since graphene and its derivatives retain properties including high surface area and rich surface chemistry. However, this work is still in the transitional stage from laboratory to industry, necessitating immense research efforts for widespread commercialization. It is expected that graphene-based hybrids will be highly useful for a number of environmental applications, particularly gas sensing and carbon dioxide conversion and storage.

Graphene has remained one of the most examined materials over the past two decades and its presumed that this will remain the case in the near future, especially in environmental applications. Since graphene was only first isolated at the start of this millennium, it requires more exploration time and scientific efforts to vigorously analyze and evaluate its different characteristics and their impact on its various applications, especially linked to environmental science, as it is still in the early stages of its development. In some cases, the use of graphene in environmental applications is similar to other families of two-dimensional materials such as carbon nanotubes and fullerenes due to their physical and chemical similarities. Therefore, the preference for graphene over other materials in future environmental applications will be based on cost-effectiveness, ease of processability, and environmental consequences. At present, graphene is still more expensive to produce than most commonly used carbon nanomaterials, i.e., activated carbon, but cheaper than single-walled carbon nanotubes; however, its production cost is expected to decrease over time with the scaling up of production processes. Considering the cost/performance of graphene in different applications compared to the traditional materials, its higher production costs could be covered by its higher performance in each application. Likewise, the health and environmental impact of graphene synthesis is expected to decrease over time with the advancements in and utilization of more environmentally friendly production procedures, particularly exfoliation-based techniques.

It is exceptionally challenging to predict the exact outlook of graphene applications since it has only been present in the ever-growing commercial market for the past few years. With its fascinating characteristics and progress in industrial-scale production, it is anticipated that the commercialization and industrialization of graphene will grow with time; however, it will be a long journey to gain market capital over other nanomaterials being used in wider applications in general and environment-related applications in particular.

We have attempted to provide a comprehensive overview of the most commonly used production processes of graphene and its environmental applications. Trend analysis of both synthesis techniques and environmental applications over the last ten years was also performed, indicating the popularity of synthesis procedures and applications with the most frequent use of graphene, making this study different from previous studies on graphene production routes and applications.

## Figures and Tables

**Table 1:** Advantages and disadvantages of various synthesis techniques for graphene.

Technique	Advantages	Disadvantages	Ref
Chemical vapour deposition	❖ High quality ❖ Large size ❖ Scalable ❖ Excellent conductivity ❖ Choice of carbon precursors	❖ Expensive ❖ Complicated procedures ❖ Un-efficient transfer process	[11]
Chemical exfoliation	❖ Low cost ❖ High yield ❖ Scalable	❖ Large number of defects ❖ Functionalised ❖ Low quality	[111]
Electrochemical exfoliation	❖ Scalable ❖ High quality ❖ Low cost ❖ Environmentally friendly ❖ Short reaction time	❖ Production of MLG ❖ Slight oxidation	[50]
Epitaxial growth	❖ No substrate transfers ❖ Seamless integration ❖ Low defects ❖ High quality	❖ High cost ❖ Uncontrolled size ❖ Multi-layered graphene	[69,91]
Liquid phase exfoliation	❖ Low cost ❖ Scalable ❖ High quality ❖ Mild experimental conditions	❖ Low yield of monolayer ❖ Time consuming ❖ Inhomogeneous flakes ❖ Small size	[112,113]
Mechanical exfoliation	❖ Monolayer production ❖ High quality ❖ Defect free ❖ Larger size	❖ Low yield ❖ Non-scalable ❖ Labour intensive	[69,114]

**Table 2:** Comparison of layer numbers, layer sizes, and electronic mobility using different graphene synthesis methods [109].

Technique	No of Layers	Size	Mobility (cm^2^v^−1^S^−1^)
Exfoliation	1 to 10+	1 mm	15,000
Thermal SiC	1 to 4	50 µm	2000
Ni-CVD	1 to 4	1 cm	3700
Cu-CVD	1	65 cm	16,000

## Data Availability

Not applicable.
